# Supplementary material for: A systematic review and meta-analysis of the diagnostic accuracy after preimplantation genetic testing for aneuploidy
Source: PLoS One. 2025 May 14;20(5):e0321859. doi: 10.1371/journal.pone.0321859 (PMC12077728; doi:10.1371/journal.pone.0321859)

# S1 Fig. Forest plots for cell line studies

## a. Positive predictive value


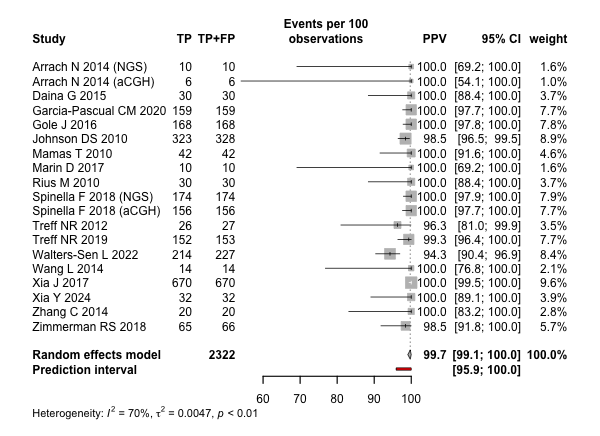


## b. Negative predictive value


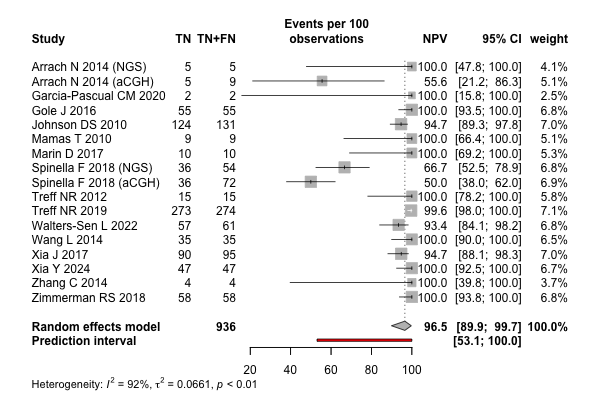


## c. Sensitivity


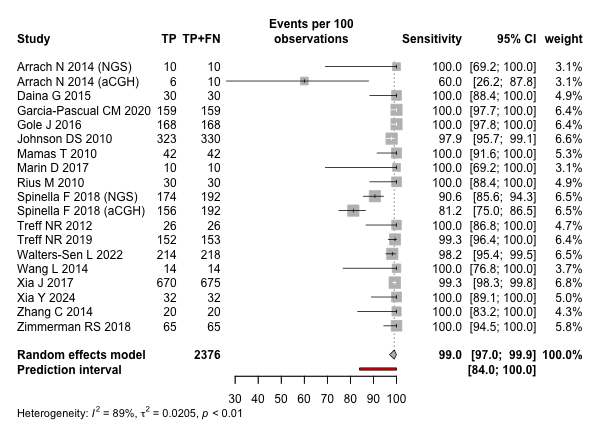


## d. Specificity


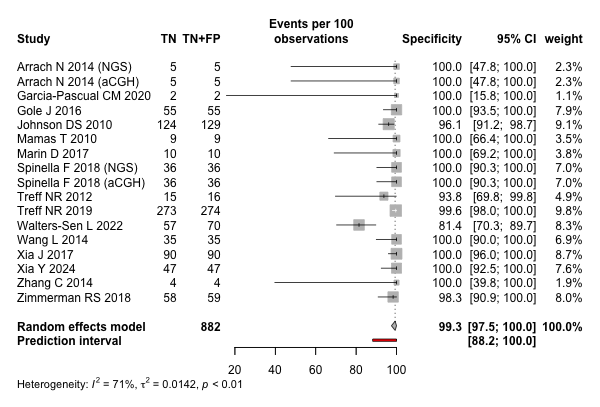

Supplement: S1 Fig — (DOCX) [file pone.0321859.s001.docx]
