# Supplementary material for: A systematic review and meta-analysis of the diagnostic accuracy after preimplantation genetic testing for aneuploidy
Source: PLoS One. 2025 May 14;20(5):e0321859. doi: 10.1371/journal.pone.0321859 (PMC12077728; doi:10.1371/journal.pone.0321859)

# S4 Figure. Forest plots for whole embryo or ICM studies: Measures of diagnostic accuracy

## Sensitivity


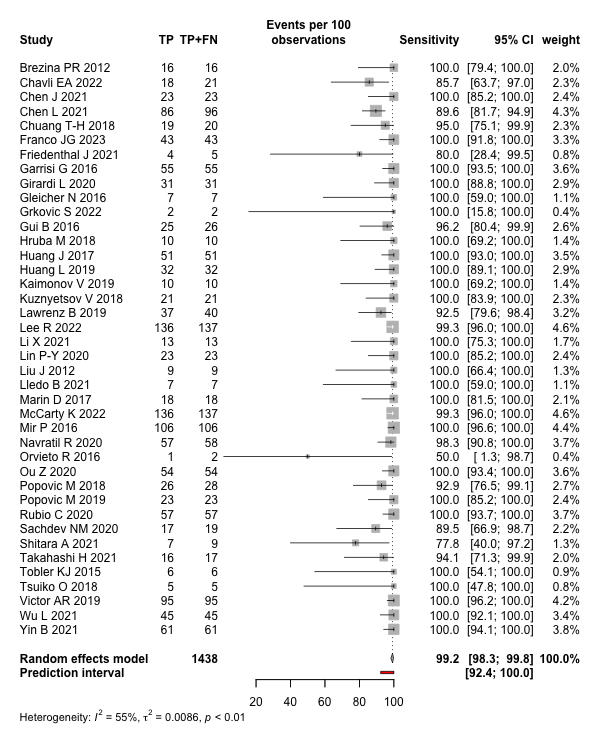


## Specificity


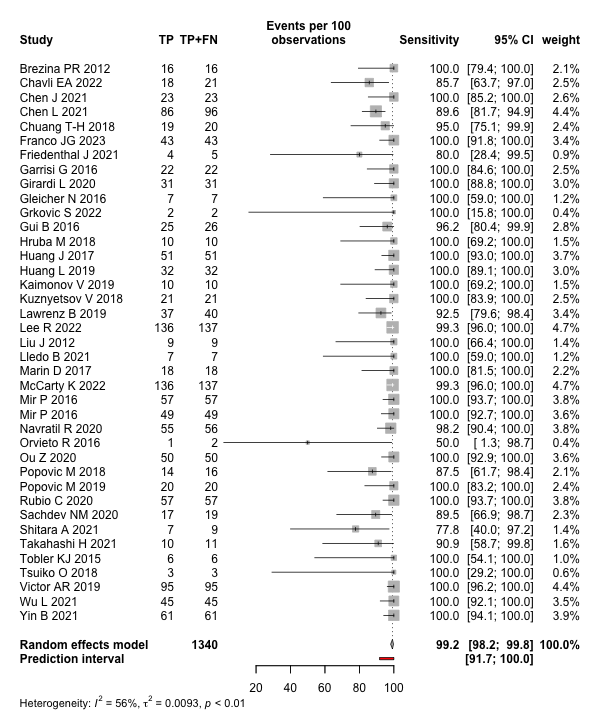


# Accuracy


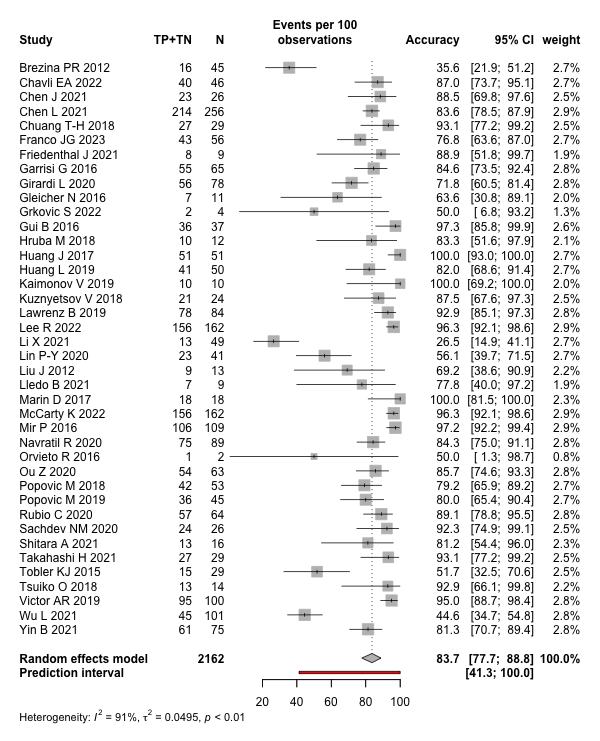

Supplement: S4 Fig — (DOCX) [file pone.0321859.s004.docx]
