# Supplementary material for: A systematic review and meta-analysis of the diagnostic accuracy after preimplantation genetic testing for aneuploidy
Source: PLoS One. 2025 May 14;20(5):e0321859. doi: 10.1371/journal.pone.0321859 (PMC12077728; doi:10.1371/journal.pone.0321859)

**S5 Fig. Forest plots for whole embryo or ICM studies subgroup analysis: NGS vs other genetic platform**

**a. Positive predictive value**


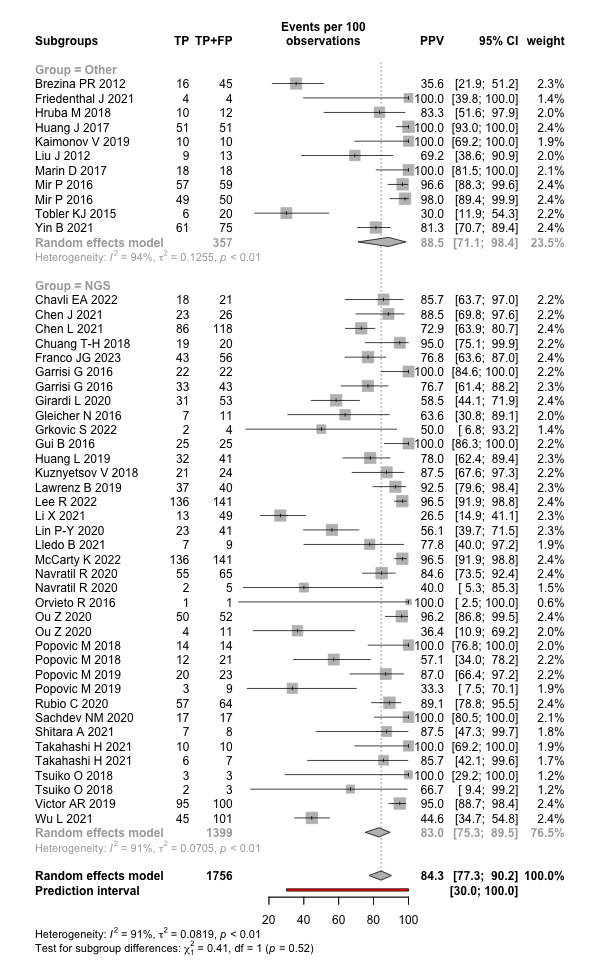


**b. Negative predictive value**


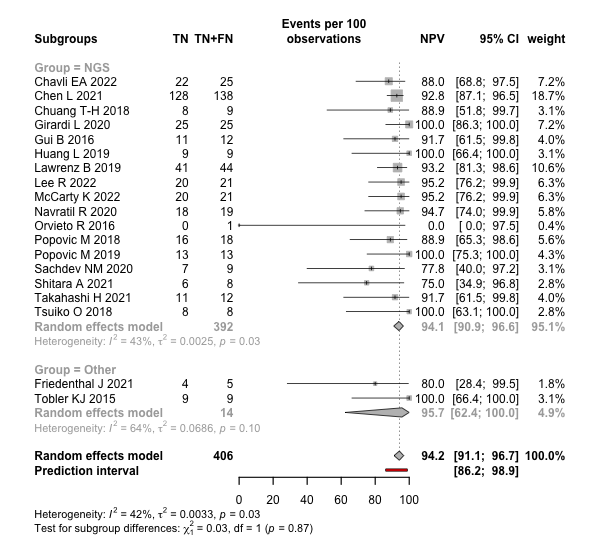

Supplement: S5 Fig — (DOCX) [file pone.0321859.s005.docx]
