# Supplementary material for: A systematic review and meta-analysis of the diagnostic accuracy after preimplantation genetic testing for aneuploidy
Source: PLoS One. 2025 May 14;20(5):e0321859. doi: 10.1371/journal.pone.0321859 (PMC12077728; doi:10.1371/journal.pone.0321859)

# S7 Fig. Forest plots for whole embryo or ICM studies subgroup analysis: Whole embryo vs ICM

## Positive predictive value


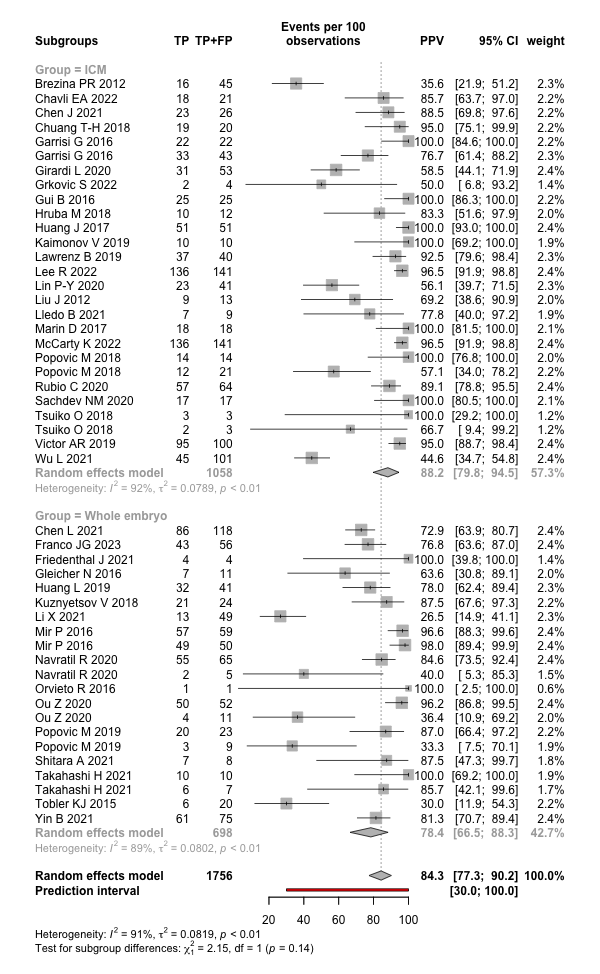


## Negative predictive value


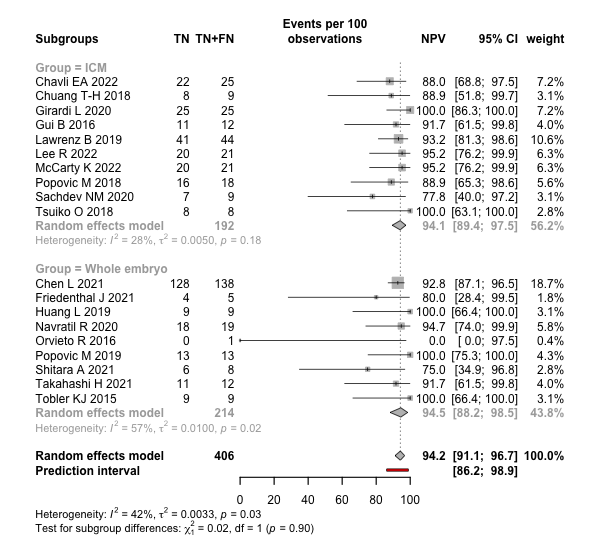

Supplement: S7 Fig — (DOCX) [file pone.0321859.s007.docx]
