# Supplementary material for: A systematic review and meta-analysis of the diagnostic accuracy after preimplantation genetic testing for aneuploidy
Source: PLoS One. 2025 May 14;20(5):e0321859. doi: 10.1371/journal.pone.0321859 (PMC12077728; doi:10.1371/journal.pone.0321859)

# S8 Fig. Forest plots for whole embryo or ICM studies subgroup analysis: Blastocyst vs cleavage stage embryos

a. Positive predictive value**
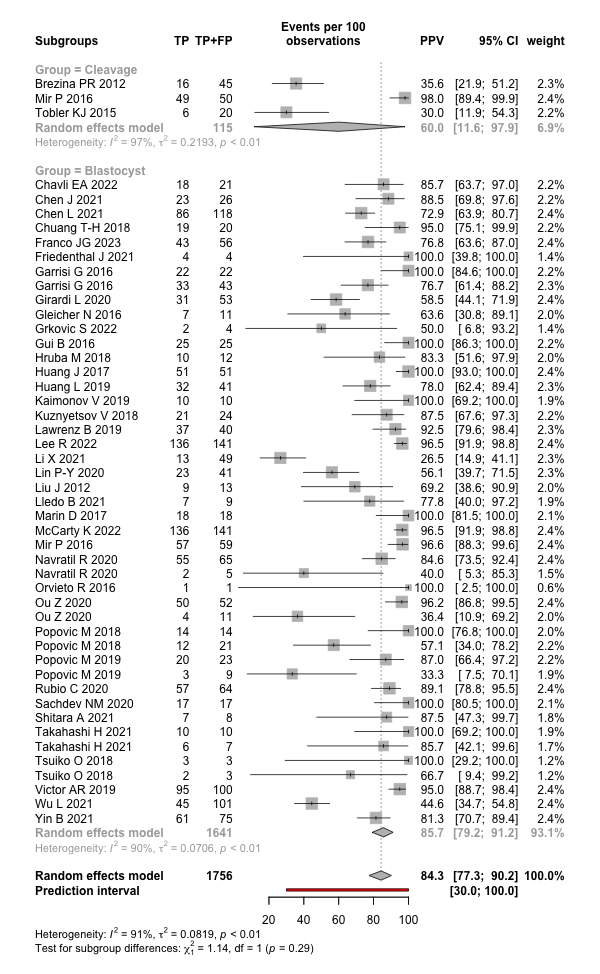
**

**b. Negative predictive value**


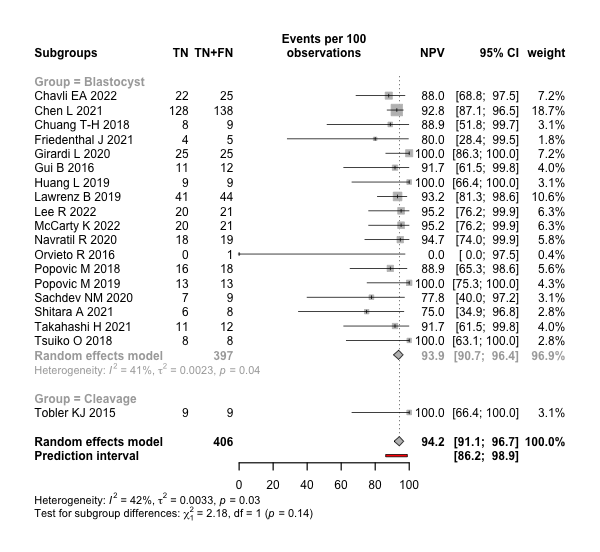

Supplement: S8 Fig — (DOCX) [file pone.0321859.s008.docx]
