# Supplementary material for: A systematic review and meta-analysis of the diagnostic accuracy after preimplantation genetic testing for aneuploidy
Source: PLoS One. 2025 May 14;20(5):e0321859. doi: 10.1371/journal.pone.0321859 (PMC12077728; doi:10.1371/journal.pone.0321859)
Supplement: S1–11 Tables — Characteristics for cell line studies, two-by-two tables all study types, and quality assessments for all study types. (DOCX) [file pone.0321859.s009.docx]

# S1 Table. Characteristics for cell line studies

| **Study** | **Publication type** | **Country** | **Cell type** | **Origin of cell line** | **Karyotype of cell lines** | **Index test: Method of aneuploidy detection** | **Method of aneuploidy detection in reference standard** |
| --- | --- | --- | --- | --- | --- | --- | --- |
| Arrach N 2014^15^ | Conference abstract | USA | Not described | Not described | 10 well-characterized cell lines (XO, XXX, XXY, XYY) with 5 control XY, XX | Array CGH and NGS | Not described |
| Daina G 2015^16^ | Full text | Spain | Fibroblasts | Coriell Cell Repository | 47,XY,+13; 48,XY,+16,+21; 48,XXX,+18; 47,XY,+15 | CGH | Not described |
| Garcia-Pascual CM 2020^17^ | Full text | Spain | Not described | Coriell Cell Repository | 9 cell lines consisting of 4-6 cells) had aneuploidies in chromosome 8, 9, 13, 18, 21, X0, XXX, XXY, XYY and normal XX and XY karyotypes (11 cell lines) | NGS | Not described |
| Gole J 2016^18^ | Conference abstract | USA | Fibroblasts | Not described | 47,XY,+9; 47,XY,+13; 47,XX,+18; 47,XY,+21; 47,XXY; 47,XYY; 46,XX; 46,XY | NGS | G-banding-derived karyotypes |
| Goodrich D 2016^19^ | Full text | USA | Fibroblasts | Coriell Cell Repository | 46,XX; 47,XY,+18; 47,XY,+15; 47,XY,+13 | qPCR and NGS | Not described |
| Goodrich D 2017^20^ | Full text | USA | Not described | Coriell Cell Repository | 46,XX,del(5)(p15.1).ish del(5)(p15.33p15.1) (D5S23-).arr 5p15.33p15.1(68519-16362247)x1); 46,XY,del(4)(p15.2).arr 4p16.3p15.2(55665- 25591051)x1 | NGS and SNP microarray | Not described |
| Johnson DS 2010^21^ | Full text | USA | Buccal cells, semen, adult blood, immortalized cell lines | Not described | T21 cells and euploid cells | SNP microarray | Metaphase karyotype |
| Mamas T 2010^22^ | Conference abstract | UK | Ovarian epithelial cells | Not described | Not described | Array CGH | Not described |
| Marin D 2017^23^ | Conference abstract | USA | Not described | Not described | 47,X?,+13/47,X?,+15; 46 XX/47,X?,+18 | NGS | Not described |
| Marin D 2017^24^ | Full text | USA | Fibroblasts | Coriell Cell Repository | 46,XY;48,XY,+16, +21 | PCR | Karyotype |
| Popovic M 2018^25^ | Full text | Belgium | Lymphocytes | Coriell Cell Repository | 47,XY,+21; 46,XX,del(18)(q22.1q23); 46,XX,del(1)(p36.33p36.22) | NGS | Not described |
| Rius M 2010^26^ | Full text | Spain | Fibroblasts | Coriell Cell Repository | 47,XY,+13; 47,XY,+15; 48,XY,+16,+21; 48,XY,+2,+21; 47,XY,+9; 48,XXX,+18 | CGH | Not described |
| Spinella F 2018^27^ | Full text | Italy | Fetal cells | Amniotic fluid | 47,XX,+18; 47,XX,+21 | Array CGH and NGS | Conventional karyotype and array CGH |
| Treff NR 2010^28^ | Full text | USA | Lymphocytes | Coriell Cell Repository | 47,XY,+9; 47,XY,+18; 45,XX,-21; 47,XY,+15; 47,XX,+8; 48,XY,+16,+21; 47,XXX; 47,XY,+13; 46,XX | SNP microarray | Not described |
| Treff NR 2012^29^ | Full text | USA | Fibroblasts and lymphocytes | Coriell Cell Repository | 47,XY,+9; 47,XY,+13; 47,XX+8[75]/46XX,+8,dic(14;21)(14qter > 14p 13::21p13 > 21qter[25]); 48,XY,+16,+21[45]/47,XY,+21[5]; 46,XY; 47,XX+21[21]/47,XX,+21,t(21;22)(q22;q13)[29]; 46,XX; 46,XY; 4,XY,+12[48]/47,XY,+12,add(13)(q34)[52] | qPCR | Conventional karyotype (G-banding) |
| Treff NR 2019^30^ | Full text | USA | Not described | Coriell Cell Repository | 49,XXXXY; 47,XYY; 47,XY,+18; 47,XY,+13; 47,XY,+15; 46,XX; 46,XY; 48,XY,+16,+21[45]47,XY,+21[5]; 47,XY,+9; 47,XX,+22; 46,X,(complex); 46,XY,der(11)t(1;11); 46,XY,t(1;11)balanced reference); 47,XXX,del(15q); 46,XY,del(11); 46,XY,del(7); 46,XX,t(1;11)(balanced reference); 47,XY,+der(13); 46,XX,del(5)(:p13 >qter); 44,XY,(complex) | SNP microarray | Not described |
| Walters-Sen L 2022^31^ | Full text | USA | Fibroblasts | Coriell Cell Repository | 47,XY,+9; 47,XY,+13; 47,XX,+18; 47,XY,+21; 47,XXY; 47,XYY; 46,XX; 46,XY | NGS | Not described |
| Wang L 2014^32^ | Full text | China | Lymphocytes | Patient's peripheral blood | 46,XX; 46,XY; 47,XY,+21 | NGS | Karyotyping by Giemsa staining and G banding |
| Warren K 2019^33^ | Conference abstract | Australia | Lymphocytes | Coriell Cell Repository | 47,XY,+15; 47,XX,+18; 48,XXY,+21; 46,XY; 46,XX | NGS | Not described |
| Xia J 2017^34^ | Conference abstract | China | Not described | Not described | Not described | NGS | Not described |
| Xia Y 2024^35^ | Full text | USA | Lymphocytes | Coriell Cell Repository | 46,XX; 46,XY; 47,XY,+9; 47,XY+18, 47,XXX; | NGS | Not described |
| Zhang C 2014^36^ | Conference abstract | USA | Not described | Not described | Segmental loss (n=6), trisomy and monosomy imbalances (n=14), normal control (n=4) | NGS | Not described |
| Zimmerman RS 2018^37^ | Full text | USA | Not described | Coriell Cell Repository | 47,XY,+22; 47,XY,+18; 47,XY,+13; 46,XX; 48XY,+16,+21; 47,XYY; 49,XXXXY; 45,XX,-21; 47,XY,+21; 47,XXX; 46,XY | NGS | Conventional G banding karyotype |

CGH: Comparative genomic hybridization; NGS: Next generation sequencing; qPCR: Quantitative polymerase chain reaction; SNP: Single nucleotide polymorphism

# S2 Table. Two-by-two table for cell line studies

| **Study** | **Number of samples tested** | **Number of samples validated to reference** | **True positive** | **True negative** | **False positive** | **False negative** |
| --- | --- | --- | --- | --- | --- | --- |
| Arrach N 2014^15^ (aCGH) | 15 | 15 | 6 | 5 | 0 | 4 |
| Arrach N 2014^15^ (NGS) | 15 | 15 | 10 | 5 | 0 | 0 |
| Daina G 2015^16^ | 30 | 30 | 30 | ¨ | ¨ | 0 |
| Garcia-Pascual CM 2020^17^ | 312 | 161 | 159 | 2 | 0 | 0 |
| Gole J 2016^18^ | 223 | 223 | 168 | 55 | 0 | 0 |
| Goodrich D 2016^19^ | 72 | ¨ | ¨ | ¨ | ¨ | ¨ |
| Goodrich D 2017^20^ | 84 | ¨ | ¨ | ¨ | ¨ | ¨ |
| Johnson DS 2010^21^ | 459 | 459 | 323 | 124 | 5 | 7 |
| Mamas T 2010^22^ | 57 | 51 | 42 | 9 | 0 | 0 |
| Marin D 2017^23^ | ¨ | ¨ | ¨ | ¨ | ¨ | ¨ |
| Marin D 2017^24^ | 20 | 20 | 10 | 10 | 0 | 0 |
| Popovic M 2018^25^ | 48 | ¨ | ¨ | ¨ | ¨ | ¨ |
| Rius M 2010^26^ | 32 | 30 | 30 | ¨ | ¨ | 0 |
| Spinella F 2018^27^ (NGS) | 228 | 228 | 174 | 36 | 0 | 18 |
| Spinella F 2018^27^ (aCGH) | 228 | 228 | 156 | 36 | 0 | 36 |
| Treff NR 2010^28^ | 99 | 27 | ¨ | ¨ | 0 | 0 |
| Treff NR 2012^29^ | 42 | 42 | 26 | 15 | 1 | 0 |
| Treff NR 2019^30^ | 446 | 427 | 152 | 273 | 1 | 1 |
| Walters-Sen L 2022^31^ | 288 | 288 | 214 | 57 | 13 | 4 |
| Wang L 2014^32^ | 49 | 49 | 14 | 35 | 0 | 0 |
| Warren K 2019^33^ | 104 | 101 | ¨ | ¨ | 0 | 0 |
| Xia J 2017^34^ | 801 | 765 | 670 | 90 | 0 | 5 |
| Xia Y 2024^35^ | 79 | 79 | 32 | 47 | 0 | 0 |
| Zhang C 2014^36^ | 24 | 24 | 20 | 4 | 0 | 0 |
| Zimmerman RS 2018^37^ | 128 | 124 | 65 | 58 | 1 | 0 |

aCGH: Array comparative genomic hybridization; NGS: Next generation sequencing

# S3 Table. Quality assessment for cell lines studies

| **Study** | **Selection risk of bias** | **Index test risk of bias** | **Index test: does conduct or interpretation differ from review question** | **Reference standard risk of bias** | **Flow risk of bias** |
| --- | --- | --- | --- | --- | --- |
| Arrach N 2014^15^ | Low risk | Unclear | Low concern | Unclear | Low risk |
| Daina G 2015^16^ | Unclear | Low risk | Low concern | Unclear | High risk |
| Garcia-Pascual CM 2020^17^ | Low risk | Unclear | Low concern | Low risk | Low risk |
| Gole J 2016^18^ | Low risk | Unclear | Low concern | Low risk | Low risk |
| Goodrich D 2016^19^ | Low risk | Low risk | Low concern | Unclear | Low risk |
| Goodrich D 2017^20^ | Low risk | High risk | Unclear | Low risk | Low risk |
| Johnson DS 2010^21^ | Unclear | Low risk | Low concern | Low risk | Low risk |
| Mamas T 2010^22^ | Unclear | Low risk | Low concern | Low risk | Low risk |
| Marin D 2017^23^ | High risk | Unclear | Unclear | Unclear | Unclear |
| Marin D 2017^24^ | Low risk | Low risk | Unclear | Unclear | Unclear |
| Popovic M 2018^25^ | Low risk | Low risk | Unclear | Unclear | Unclear |
| Rius M 2010^26^ | High risk | Unclear | Low concern | Unclear | Low risk |
| Spinella F 2018^27^ | Low risk | Low risk | Low concern | Unclear | Low risk |
| Treff NR 2010^28^ | Low risk | Unclear | Low concern | Low risk | Low risk |
| Treff NR 2012^29^ | Low risk | Low risk | Low concern | Low risk | Low risk |
| Treff NR 2019^30^ | Low risk | Unclear | Low concern | Low risk | Low risk |
| Walters-Sen L 2022^31^ | Low risk | Low risk | Unclear | Low risk | Low risk |
| Wang L 2014^32^ | Low risk | Unclear | Low concern | Low risk | Low risk |
| Warren K 2019^33^ | Low risk | Unclear | Low concern | Low risk | Low risk |
| Xia J 2017^34^ | Low risk | Unclear | Low concern | Low risk | Low risk |
| Xia Y 2024^35^ | Low risk | Low risk | Low concern | Unclear | Unclear |
| Zhang C 2014^36^ | Low risk | Unclear | Unclear | Low risk | Low risk |
| Zimmerman RS 2018^37^ | Low risk | Low risk | Low concern | Low risk | Low risk |

# S4 Table. Two-by-two table for whole embryo or ICM studies – euploid/aneuploid embryos

| **Study** | **Number of embryos validated to reference** | **True positive** | **True negative** | **False positive** | **False negative** |
| --- | --- | --- | --- | --- | --- |
| Brezina PR 2012^38^ | 45 | 16 | - | 29 | - |
| Chavli EA 2022^39^ | 46 | 18 | 22 | 3 | 3 |
| Chen J 2021^40^ | 26 | 23 | - | 3 | - |
| Chen L 2021^41^ | 256 | 86 | 128 | 32 | 10 |
| Chuang T-H 2018^42^ | 29 | 19 | 8 | 1 | 1 |
| Franco JG 2023^43^ | 56 | 43 | - | 13 | - |
| Friedenthal J 2021^44^ | 9 | 4 | 4 | 0 | 1 |
| Garrisi G 2016^45^ | 22 | 22 | - | 0 | - |
| Girardi L 2020^46^ | 78 | 31 | 25 | 22 | 0 |
| Gleicher N 2016^7^ | 11 | 7 | - | 4 | - |
| Grkovic S 2022^47^ | 4 | 2 | - | 2 | - |
| Gui B 2016^48^ | 37 | 25 | 11 | 0 | 1 |
| Hruba M 2018^49^ | 12 | 10 | - | 2 | - |
| Huang J 2017^50^ | 51 | 51 | - | 0 | - |
| Huang L 2019^51^ | 50 | 32 | 9 | 9 | 0 |
| Kaimonov V 2019^52^ | 10 | 10 | - | 0 | - |
| Kuznyetsov V 2018^53^ | 24 | 21 | - | 3 | - |
| Lawrenz B 2019^54^ | 84 | 37 | 41 | 3 | 3 |
| Lee R 2022^55^ | 162 | 136 | 20 | 5 | 1 |
| Liu J 2012^58^ | 13 | 9 | - | 4 | - |
| Lledo B 2021^59^ | 9 | 7 | - | 2 | - |
| Marin D 2017^24^ | 18 | 18 | - | 0 | - |
| McCarty K 2022^60^ | 162 | 136 | 20 | 5 | 1 |
| Mir P 2016^61^ | 59 | 57 | - | 2 | - |
| Navratil R 2020^62^ | 84 | 55 | 18 | 10 | 1 |
| Orvieto R 2016^6^ | 2 | 1 | - | - | 1 |
| Ou Z 2020^63^ | 52 | 50 | - | 2 | - |
| Popovic M 2018^25^ | 32 | 14 | 16 | 0 | 2 |
| Popovic M 2019^64^ | 36 | 20 | 13 | 3 | 0 |
| Rubio C 2020^65^ | 64 | 57 | - | 7 | - |
| Sachdev NM 2020^66^ | 26 | 17 | 7 | 0 | 2 |
| Shitara A 2021^67^ | 16 | 7 | 6 | 1 | 2 |
| Takahashi H 2021^68^ | 22 | 10 | 11 | 0 | 1 |
| Tobler KJ 2015^69^ | 29 | 6 | 9 | 14 | 0 |
| Tsuiko O 2018^70^ | 11 | 3 | 8 | 0 | 0 |
| Victor AR 2019^71^ | 100 | 95 | - | 5 | - |
| Wu L 2021^72^ | 101 | 45 | - | 56 | - |
| Yin B 2021^73^ | 75 | 61 | - | 14 | - |
|  |  |  |  |  |  |

# S5 Table. Two-by-two table for whole embryo or ICM studies – mosaic embryos

| **Study** | **Number of embryos validated to reference** | **True positive** | **True negative** | **False positive** | **False negative** |
| --- | --- | --- | --- | --- | --- |
| Garrisi G 2016^45^ | 43 | 33 | - | 10 | - |
| Li X 2021^56^ | 49 | 13 |  | 36 |  |
| Lin P-Y 2020^57^ | 41 | 23 | - | 18 | - |
| Navratil R 2020^62^ | 5 | 2 | - | 3 | - |
| Ou Z 2020^63^ | 11 | 4 | - | 7 | - |
| Popovic M 2018^25^ | 21 | 12 | - | 9 | - |
| Popovic M 2019^64^ | 9 | 3 | - | 6 | - |
| Takahashi H 2021^68^ | 7 | 6 | - | 1 | - |
| Tsuiko O 2018^70^ | 3 | 2 | - | 1 | - |

# S6 Table. Quality assessment whole embryo or ICM studies

| **Study** | **Patient selection risk of bias** | **Index test risk of bias** | **Index test: Does conduct or interpretation differ from review question** | **Reference standard risk of bias** | **Flow risk of bias** |
| --- | --- | --- | --- | --- | --- |
| Brezina PR 2012^38^ | Unclear | Low risk | Low concern | Unclear | High risk |
| Chavli EA 2022^39^ | Unclear | Low risk | Low concern | Low risk | Low risk |
| Chen J 2021^40^ | High risk | Low risk | Low concern | Low risk | Low risk |
| Chen L 2021^41^ | High risk | Low risk | Low concern | Unclear | Low risk |
| Chuang T-H 2018^42^ | Low risk | Unclear | Low concern | Unclear | Low risk |
| Franco JG 2023^43^ | Unclear | Low risk | Unclear | Low risk | Low risk |
| Friedenthal J 2021^44^ | Unclear | Unclear | Unclear | Unclear | Low risk |
| Garrisi G 2016^45^ | Unclear | Unclear | Low concern | Unclear | Unclear |
| Girardi L 2020^46^ | Low risk | Low risk | Low concern | Low risk | Low risk |
| Gleicher N 2016^7^ | Unclear | Low risk | Low concern | Unclear | Unclear |
| Grkovic S 2022^47^ | Unclear | Low risk | Low concern | Unclear | High risk |
| Gui B 2016^48^ | High risk | Low risk | Low concern | Low risk | Unclear |
| Hruba M 2018^49^ | Unclear | Unclear | Low concern | Unclear | Low risk |
| Huang J 2017^50^ | High risk | Low risk | Low concern | Unclear | Low risk |
| Huang L 2019^51^ | Unclear | Low risk | Low concern | Low risk | Low risk |
| Kaimonov V 2019^52^ | Unclear | Low risk | Low concern | Unclear | Low risk |
| Kuznyetsov V 2018^53^ | Unclear | Low risk | Low concern | Low risk | Low risk |
| Lawrenz B 2019^54^ | Low risk | Unclear | Low concern | Unclear | Low risk |
| Lee R 2022^55^ | Unclear | Low risk | Low concern | Low risk | Low risk |
| Li X 2021^56^ | High risk | Low risk | Unclear | Unclear | Low risk |
| Lin P-Y 2020^57^ | Low risk | Low risk | Low concern | Low risk | Unclear |
| Liu J 2012^58^ | High risk | Low risk | Low concern | Unclear | High risk |
| Lledo B 2021^59^ | Unclear | Low risk | Low concern | Low risk | High risk |
| Marin D 2017^24^ | High risk | Unclear | Unclear | Unclear | Unclear |
| McCarty K 2022^60^ | Low risk | Low risk | Low concern | Low risk | Unclear |
| Mir P 2016^61^ | Unclear | Low risk | Low concern | Low risk | Low risk |
| Navratil R 2020^62^ | Unclear | Low risk | Low concern | Low risk | Low risk |
| Orvieto R 2016^6^ | Unclear | High risk | Low concern | Unclear | High risk |
| Ou Z 2020^63^ | High risk | Low risk | Unclear | Low risk | Low risk |
| Popovic M 2018^25^ | Unclear | Low risk | Low concern | Unclear | Low risk |
| Popovic M 2019^64^ | Unclear | Low risk | Low concern | Unclear | High risk |
| Rubio C 2020^65^ | Low risk | Low risk | Low concern | Low risk | High risk |
| Sachdev NM 2020^66^ | Unclear | Low risk | Low concern | High risk | High risk |
| Shitara A 2021^67^ | Unclear | Unclear | Low concern | Unclear | High risk |
| Takahashi H 2021^68^ | Low risk | Low risk | Low concern | Low risk | Low risk |
| Tobler KJ 2015^69^ | Unclear | Unclear | High concern | Unclear | High risk |
| Tsuiko O 2018^70^ | Unclear | Unclear | Unclear | Unclear | Unclear |
| Victor AR 2019^71^ | Unclear | Low risk | Low concern | Low risk | Low risk |
| Wu L 2021^72^ | Unclear | Unclear | Low concern | Low risk | Low risk |
| Yin B 2021^73^ | Unclear | Low risk | Low concern | Low risk | Low risk |

# S7 Table. Two-by-two table for pregnancy outcomes studies: euploid embryos

| **Study** | **Number of embryos transferred** | **Number of embryos validated** | **Number of pregnancies** | **Number of misdiagnoses** |
| --- | --- | --- | --- | --- |
| Chamayou S 2015^77^ | 7 | 4 | 4 | 0 |
| Chen D 2020^78^ | 16 | 7 | 11 | 0 |
| Daina G 2015^16^ | 11 | 2 | 2 | 0 |
| Friedenthal J 2020^4^ | 1997 | ¨ | 1335 | 19 |
| Hu X 2024^81^ | 19 | 8 | 15 | 0 |
| Huang C 2022^82^ | 3 | 3 | 3 | 0 |
| Huang J 2015^83^ | 7 | 5 | 5 | 0 |
| Katz-Jaffe M 2023^84^ | 3992 | - | - | 22 |
| Kim JG 2021^85^ | 6671 | 281 | 4515 | 39 |
| Klimczak AM 2020^86^ | ¨ | 12 | ¨ | 1 |
| Luo KL 2015^87^ | ¨ | 7 | 60 | 0 |
| Ma GC 2016^88^ | 30 | 3 | 17 | 0 |
| Ma X 2021^89^ | 215 | 168 | 179 | 0 |
| Maxwell SM 2016^5^ | 76 | 20 | 76 | 4 |
| Morales Sabater R 2023^90^ | - | 115 | - | 0 |
| Mykytenko D 2018^91^ | ¨ | 20 | ¨ | 2 |
| Ou Z 2022^92^ | 29 | 22 | 22 | 0 |
| Ruttanajit T 2016^95^ | 12 | 4 | 5 | 0 |
| Satirapod C 2019^96^ | ¨ | 8 | 9 | 0 |
| Scott RT 2012^97^ | 133 | 58 | 58 | 0 |
| Tan Y 2014^100^ | 406 | 99 | 199 | 0 |
| Tao X 2020^101^ | 120 | 120 | 120 | 11 |
| Tiegs AW 2016^102^ | 579 | ¨ | 356 | 5 |
| Tiegs AW 2021^103^ | 312 | ¨ | 256 | 0 |
| Treff NR 2011^104^ | 20 | 4 | 10 | 0 |
| Vesela K 2019^105^ | ¨ | 32 | ¨ | 1 |
| Volozonoka L 2018^107^ | 6 | 3 | 5 | 0 |
| Wang J 2018^108^ | 10 | 3 | 7 | 0 |
| Wang J 2023^109^ | - | 6 | 15 | 0 |
| Wang Y 2021^110^ | 8 | 3 | 4 | 0 |
| Wang Y 2023^111^ | 6 | 3 | 4 | 0 |
| Wells D 2009^112^ | 42 | 12 | 36 | 0 |
| Werner MD 2014^113^ | 4974 | ¨ | 2976 | 10 |
| Wiltshire AM 2021^114^ | ¨ | 67 | ¨ | 8 |
| Yang J 2021^115^ | 9 | 1 | 3 | 0 |
| Yao Z 2023^116^ | - | 16 | - | 0 |
| Zhai F 2022^117^ | 73 | 18 | 33 | 0 |
| Zhang L 2019^118^ | 370 | 207 | 271 | 0* |
| Zhang S 2017^119^ | 13 | 11 | 11 | 0 |
| Zhang S 2019^120^ | 6 | 4 | 4 | 0 |
| Zhang S 2021^121^ | 12 | 9 | 9 | 0 |
| Zhou Z 2018^123^ | 12 | 2 | 6 | 0 |

*Reanalyzed aCGH plots and redefined mosaicism of previously classified euploid transferred embryos

# S8 Table. Two-by-two table for pregnancy outcomes studies: aneuploid embryos

| **Study** | **Number of embryos transferred** | **Number of embryos validated** | **Number of pregnancies** | **Number of misdiagnoses** |
| --- | --- | --- | --- | --- |
| Barad DH 2022^75^ | 21 | 8 | 9 | 3 |
| Gleicher N 2016^7^ | 9 | 5 | 6 | 5 |
| Scott RT 2012^97^ | 99 | 6 | 6 | 4 |
| Tiegs AW 2021^103^ | 102 | 5 | 24 | 0 |

# S9 Table. Two-by-two table for pregnancy outcomes studies: non-selection studies

| **Study** | **Number of embryos transferred** | **Number of embryos validated** | **Number of pregnancies** | **Number of misdiagnoses** |
| --- | --- | --- | --- | --- |
| Scott RT 2012^97^ | 255 | 64 | 72 | 4 |
| Shen X 2019^198^ | 33 | 31 | 92 | ¨ |
| Tiegs AW 2021^103^ | 484 | ¨ | 346 | 1 |

# S10 Table. Two-by-two table for pregnancy outcomes studies: mosaic studies

| **Study** | **Number of embryos transferred** | **Number of embryos validated** | **Number of pregnancies** | **Number of misdiagnoses** |
| --- | --- | --- | --- | --- |
| Aharon D 2022^74^ | 27 | 5 | 15 | 5 |
| Barad DH 2022^75^ | 35 | 9 | 11 | 8 |
| Besser AG 2019^76^ | 40 | 6 | 18 | 6 |
| Fernandez Sanguino A 2022^79^ | 8 | 5 | 5 | 5 |
| Gao Y 2022^80^ | - | 10 | 10 | 7 |
| Lin P-Y 2020^57^ | 108 | 46 | 56 | 46 |
| Morales Sabater R 2023^90^ | - | 10 | 172 | 10 |
| Pozzoni M 2022^93^ | - | 24 | 39 | 22^**^ |
| Rubino P 2018^94^ | 33 | 17 | 20 | 17 |
| Spinella F 2018^27^ | 78 | 24 | 37 | 24 |
| Spinella F 2023^99^ | 2045 | 550 | 670 | 445 |
| Victor AR 2019^106^ | 100 | 14 | 49 | 0 |
| Yao Z 2023^116^ | - | 1 | - | 1 |
| Zhang YX 2020^122^ | 137 | 3 | 55 | 3 |

*Invasive testing or postnatal karyotype was conducted on 10 pregnancies/neonates. Mosaicism did not match prenatal testing or postnatal testing

**Mosaicism did not match CVS/amniocentesis results in 2 patients (CPM T15 after M6 transfer; fetal Mdup 6pm after mosaic del 10p)

# S11 Table. Quality assessment pregnancy outcomes studies

| **Study** | **Patient selection risk of bias** | **Index test risk of bias** | **Index test: Does conduct or interpretation differ from review question** | **Reference standard risk of bias** | **Flow risk of bias** |
| --- | --- | --- | --- | --- | --- |
| Aharon D 2022^74^ | Unclear | Low risk | Low concern | Low risk | High risk |
| Barad DH 2022^75^ | High risk | Unclear | Unclear | Unclear | High risk |
| Besser AG 2019^76^ | Unclear | Low risk | Low concern | Unclear | Unclear |
| Chamayou S 2015^77^ | Unclear | Unclear | Unclear | Unclear | High risk |
| Chen D 2020^78^ | High risk | Low risk | Low concern | Unclear | High risk |
| Daina G 2015^16^ | Unclear | Low risk | Low concern | Unclear | High risk |
| Fernandez Sanguino A^79^ | Unclear | Low risk | Low concern | Unclear | High risk |
| Friedenthal J 2020^4^ | Low risk | Low risk | Low concern | Unclear | High risk |
| Gao Y 2022^80^ | Unclear | Low risk | Low concern | Low risk | High risk |
| Gleicher N 2016^7^ | Unclear | Low risk | Low concern | Unclear | Unclear |
| Hu X 2024^81^ | Unclear | Low risk | Low concern | Unclear | High risk |
| Huang C 2022^82^ | High risk | Low risk | Low concern | High risk | Low risk |
| Huang J 2015^83^ | High risk | Low risk | Low concern | Unclear | High risk |
| Katz-Jaffe M 2023^84^ | Unclear | Low risk | Low concern | Unclear | High risk |
| Kim JG 2021^85^ | Unclear | Unclear | Unclear | Unclear | Unclear |
| Klimczak AM 2020^86^ | Low risk | Unclear | Unclear | Unclear | High risk |
| Lin P-Y 2020^57^ | Low risk | Low risk | Low concern | Low risk | Unclear |
| Luo KL 2015^87^ | Low risk | Low risk | Low concern | Unclear | High risk |
| Ma GC 2016^88^ | Low risk | Low risk | Low concern | Unclear | High risk |
| Ma X 2021^89^ | High risk | Low risk | Low concern | High risk | High risk |
| Maxwell SM 2016^5^ | Unclear | Low risk | Low concern | High risk | High risk |
| Morales Sabater R 2023^90^ | High risk | Low risk | Low concern | High risk | High risk |
| Mykytenko D 2018^91^ | Unclear | Low risk | Low concern | Low risk | High risk |
| Ou Z 2022^92^ | High risk | Low risk | Low concern | Unclear | High risk |
| Pozzoni M 2022^93^ | High risk | Low risk | Low concern | Unclear | High risk |
| Rubino P 2018^94^ | Unclear | Low risk | Low concern | Unclear | High risk |
| Ruttanajit T 2016^95^ | Unclear | Low risk | Low concern | Unclear | High risk |
| Satirapod C 2019^96^ | Low risk | Low risk | Low concern | Unclear | High risk |
| Scott RT 2012^97^ | Low risk | Low risk | Unclear | Unclear | High risk |
| Shen X 2019^98^ | Unclear | High risk | Low concern | High risk | High risk |
| Spinella F 2018^27^ | Unclear | Low risk | Low concern | Unclear | High risk |
| Spinella F 2023^99^ | High risk | Low risk | Low concern | High risk | High risk |
| Tan Y 2014^100^ | High risk | Unclear | Low concern | Unclear | High risk |
| Tao X 2020^101^ | Unclear | Low risk | Unclear | Unclear | Low risk |
| Tiegs AW 2016^102^ | Unclear | Low risk | Low concern | High risk | High risk |
| Tiegs AW 2021^103^ | Low risk | Low risk | Low concern | High risk | High risk |
| Treff NR 2011^104^ | High risk | Low risk | Low concern | Low risk | High risk |
| Vesela K 2019^105^ | Unclear | Low risk | Low concern | Low risk | Unclear |
| Victor AR 2019^106^ | Unclear | High risk | Low concern | Unclear | High risk |
| Volozonoka L 2018^107^ | Low risk | Unclear | Low concern | Unclear | High risk |
| Wang J 2018^108^ | High risk | Low risk | Low concern | Unclear | High risk |
| Wang J 2023^109^ | Low risk | Low risk | Low concern | Unclear | High risk |
| Wang Y 2021^110^ | Unclear | Low risk | Low concern | High risk | High risk |
| Wang Y 2023^111^ | Low risk | Low risk | Low concern | Unclear | High risk |
| Wells D 2009^112^ | Unclear | Low risk | Low concern | Unclear | High risk |
| Werner MD 2014^113^ | Low risk | Low risk | Low concern | High risk | High risk |
| Wiltshire AM 2021^114^ | Low risk | Unclear | Unclear | Low risk | High risk |
| Yang J 2021^115^ | Unclear | Low risk | Low concern | Unclear | High risk |
| Yao Z 2023^116^ | Unclear | Low risk | Low concern | Low risk | High risk |
| Zhai F 2022^117^ | High risk | Low risk | Low concern | Unclear | High risk |
| Zhang L 2019^118^ | High risk | Low risk | Low concern | Unclear | High risk |
| Zhang S 2017^119^ | High risk | Low risk | Unclear | Low risk | High risk |
| Zhang S 2019^120^ | Low risk | Unclear | Low concern | Unclear | High risk |
| Zhang S 2021^121^ | Unclear | Unclear | Low concern | Unclear | High risk |
| Zhang YX 2020^122^ | High risk | Low risk | Unclear | Unclear | High risk |
| Zhou Z 2018^123^ | High risk | Low risk | Low concern | Unclear | High risk |
