# Supplementary material for: A systematic review and meta-analysis of the diagnostic accuracy after preimplantation genetic testing for aneuploidy
Source: PLoS One. 2025 May 14;20(5):e0321859. doi: 10.1371/journal.pone.0321859 (PMC12077728; doi:10.1371/journal.pone.0321859)
Supplement: S1 File — (DOC) [file pone.0321859.s010.doc]

**S1 File. Medline search strategy**

--------------------------------------------------------------------------------

1 exp Aneuploidy/

2 (aneuploid* or aneuploid cell* or monosomy or monosomies or tetrasomy or tetrasomies or trisomy or trisomies).ti,ab,kf.

3 (chromosom* adj3 (abnormalit* or anomal* or aberration* or irregularit*)).ti,ab,kf.

4 or/1-3

5 Preimplantation Diagnosis/

6 ((preimplantation or pre-implantation) adj3 (testing or screening or diagnos* or aneuploid* or biops*)).ti,ab,kf.

7 (PGT-A or PGT or PGS or niPGT-A or niPGTA or miPGT-A or miPGTA).ti,ab,kf.

8 ((blastocyte* or blastocyst* or embryo*) adj3 (testing or screening or diagnos* or biops*)).ti,ab,kf.

9 ((trophectoderm or trophoblast*) adj3 (testing or screening or biops*)).ti,ab,kf

10 or/5-9

11 exp "Sensitivity and Specificity"/

12 "Predictive Value of Tests"/

13 (sensitivity or specificity or "sensitivity and specificity" or positive predictive value* or negative predictive value* or clinical decision making or clinical decision-making or valid* or reliab* or evaluation* or reproducibility of result* or implication* or discordance or concordance or consistenc* or false positive* or false negative* or under the curve or kappa agreement* or error* or success*).ti,ab,kf.

14 or/11-13

15 4 and 10 and 14

16 from 15 keep 1-818
