# Supplementary material for: A systematic review and meta-analysis of the diagnostic accuracy after preimplantation genetic testing for aneuploidy
Source: PLoS One. 2025 May 14;20(5):e0321859. doi: 10.1371/journal.pone.0321859 (PMC12077728; doi:10.1371/journal.pone.0321859)
Supplement: S2 File — (DOCX) [file pone.0321859.s011.docx]

# S2 File. Excluded studies with reasons for exclusion

| Title | Authors | Published Year | Journal | Volume | Issue | Pages | DOI | Reason for Exclusion |
| --- | --- | --- | --- | --- | --- | --- | --- | --- |
| Array-comparative genomic hybridization (array-CGH): The first report of its clinical application for preimplantation genetic screening (PGS) in Iran | Aflatoonian, A.; Karimzadeh Meybodi, M. A.; Yousefnejad, F.; Taheripanah, R.; Hosseini, E.; Nikmard, F.; Amjadi, F. S.; Hamidi, D.; Kariminejad, R.; Aflatoonian, B.; Aflatoonian, R. | 2017 | International Journal of Reproductive BioMedicine | 15 | 4(SUPPL. 1) | 7 |  | Outcomes are unclear or not relevant |
| Cytogenetic analysis of human blastocysts with the use of FISH, CGH and aCGH: Scientific data and technical evaluation | Alfarawati, S.; Goodall, N.; Gordon, T.; Gunner, J.; Daphnis, D.; Wells, D.; Fragouli, E. | 2010 | Human Reproduction | 25 | SUPPL. 1 | i41-i42 | 10.1093/humrep/de.25.s1.30 | Duplicate |
| Individualised luteal phase support in artificially prepared frozen embryo transfer cycles based on serum progesterone levels: a prospective cohort study | Alvarez, M.; Gaggiotti-Marre, S.; Martinez, F.; Coll, L.; Garcia, S.; Gonzalez-Foruria, I.; Rodriguez, I.; Parriego, M.; Polyzos, N. P.; Coroleu, B. | 2021 | Human Reproduction | 36 | 6 | 1552-1560 | https://dx.doi.org/10.1093/humrep/deab031 | Outcomes are unclear or not relevant |
| Concordance rates among multiple trophectoderm, samples and ICM remain high proving the efficacy of NGS for, preimplantation genetic screening | Anderson, R.; Whitney, J. B.; Rios, C. M.; Jones, A. E.; Harutunian, A.; Schiewe, M. C. | 2018 | Human Reproduction | 33 | SUPPL. 1 | i121 | 10.1093/humrep/33.Supplement_1.1 | Inadequate information |
| Fluorescence in situ hybridization analysis of two blastomeres from day 3 frozen-thawed embryos followed by analysis of the remaining embryo on day 5 | Baart, E. B.; Van Opstal, D.; Los, F. J.; Fauser, B. C.; Martini, E. | 2004 | Human Reproduction | 19 | 3 | 685-693 | 10.1093/humrep/deh094 | Use of FISH |
| The clinical significance of segmental aneuploidy in human oocytes and preimplantation embryos | Babariya, D.; Fragouli, E.; Alfarawati, S.; Spaeth, K.; Raberi, A.; Taylor, S.; Kubikova, N.; Wells, D. | 2016 | Human Reproduction | 31 | SUPPL. 1 | i13-i14 | http://dx.doi.org/10.1093/humrep/31.Supplement_1.1 | Wrong study design |
| Haploseek: a 24-hour all-in-one method for preimplantation genetic diagnosis (PGD) of monogenic disease and aneuploidy | Backenroth, D.; Zahdeh, F.; Kling, Y.; Peretz, A.; Rosen, T.; Kort, D.; Zeligson, S.; Dror, T.; Kirshberg, S.; Burak, E.; Segel, R.; Levy-Lahad, E.; Zangen, D.; Altarescu, G.; Carmi, S.; Zeevi, D. A. | 2019 | Genetics in Medicine | 21 | 6 | 1390-1399 | 10.1038/s41436-018-0351-7 | Wrong study design |
| Evaluating the plausibility of euploid embryos transfer on day-5 by reanalysis of day-3 single aneuploid embryos: a case series | Bazrgar, M.; Kariminejad R.; Eftekhari-Y, P.; Gourabi, H. | 2024 | Journal of Reproduction& Infertility | 25 | 1 | 56-59 | 10.18502/jri.v25i1.15200 | Wrong comparator |
| Four years of prospective mosaic embryo transfer: a single center's experience | Besser, A. G.; Blakemore, J. K.; Del Buono, E. J.; McCaffrey, C.; McCulloh, D. H.; Grifo, J. A. | 2019 | Fertility and Sterility | 112 | 3(SUPPL.) | e230 | 10.1016/j.fertnstert.2019.07.712 | Inadequate information |
| Pregnancy outcomes following in vitro fertilization frozen embryo transfer (IVF-FET) with or without preimplantation genetic testing for aneuploidy (PGT-A) in women with recurrent pregnancy loss (RPL): a SART-CORS study | Bhatt, S. J.; Marchetto, N. M.; Roy, J.; Morelli, S. S.; McGovern, P. G. | 2021 | Human Reproduction | 36 | 8 | 2339-2344 | https://dx.doi.org/10.1093/humrep/deab117 | Outcomes are unclear or not relevant |
| Detection of >=1Mb microdeletions and microduplications in a single cell using custom oligonucleotide arrays | Bi, W.; Breman, A.; Shaw, C. A.; Stankiewicz, P.; Gambin, T.; Lu, X.; Cheung, S. W.; Jackson, L. G.; Lupski, J. R.; Den Veyver, I. B. V.; Beaudet, A. L. | 2012 | Prenatal Diagnosis | 32 | 1 | 10-20 | 10.1002/pd.2855 | Wrong study design |
| Detection of segmental aneuploidy and mosaicism in preimplantation embryo model by next generation sequencing methodologies | Biricik, A.; Cotroneo, E.; Bono, S.; Surdo, M.; Minasi, M. G.; Cursio, E.; Greco, E.; Fiorentino, F.; Spinella, F. | 2018 | Reproductive BioMedicine Online | 36 | SUPPL. 1 | e15 | http://dx.doi.org/10.1016/j.rbmo.2017.10.036 | Duplicate |
| Increased efficiency on trophectoderm analysis during preimplantation genetic diagnosis (PGD) | Buchholz, T.; Heiliger, K. J.; Gutknecht, D.; Adelfalk, C.; Eder, A.; Bals-Pratsch, M. | 2013 | Fertility and Sterility | 100 | 3(SUPPL.) | S204-S205 | 10.1016/j.fertnstert.2013.07.1371 | Outcomes are unclear or not relevant |
| Promising transfer and pregnancy rates utilizing consecutive "freeze all"-cycles and trophectoderm aneuploidy screening | Bug, S.; Gros, B.; Solfrank, B.; Stecher, M.; Pricelius, J.; Nevinny-Stickel-Hinzpeter, C.; Noss, U. | 2014 | Human Reproduction | 29 | SUPPL. 1 | i174-i175 | 10.1093/humrep/29.Supplement_1.1 | Outcomes are unclear or not relevant |
| The necessity to discuss combined PGD/PGS and the demonstration of its feasibility with currently available methods | Bug, S.; Moser, T.; Solfrank, B.; Pricelius, J.; Laitinen-Forsblom, P. J.; Nevinny-Stickel-Hinzpeter, C. | 2014 | Medizinische Genetik | 26 | 1 | 190 |  | Wrong study design |
| Checking on mosaicism in day 5-blastocysts first results from a microarray based diagnostic setting | Bug, S.; Stecher, M.; Pricelius, J.; Gros, B.; Noss, U.; Nevinny-Stickel-Hinzpeter, C. | 2012 | Reproductive BioMedicine Online | 24 | SUPPL. 2 | S56 | 10.1016/S1472-6483%2812%2960245-4 | Inadequate information |
| Introducing direct CGG repeat analysis in preimplantation genetic diagnosis (PGD) for fragile X syndrome: An overview of clinical outcomes for 116 patients | Cabey, R.; Nusblat, D.; Gay, J.; Armenti, E.; Chu, B.; Goodall, N.; Prates, R.; Konstantinidis, M. | 2018 | Human Reproduction | 33 | SUPPL. 1 | i422 | 10.1093/humrep/33.Supplement_1.1 | Inadequate information |
| Majority of transferred mosaic embryos developed healthy live births revealed by a preclinical study using embryonic morphology assessment and noninvasive PGT-A on cell-free DNA in blastocoel fluid | Cai, L.; Zeng, Q.; Wu, W.; Shen, J.; Wu, B-L.; Wang, DW.; Cui, Y.; Liu, J. | 2022 | Journal of Assisted Reproduction and Genetics | 39 | 11 | 2483-2504 | 10.1007/s10815-022-02651-5 | Wrong comparartor |
| Clinical validity and utility of preconception expanded carrier screening for the management of reproductive genetic risk in IVF and general population | Capalbo, A.; Fabiani, M.; Caroselli, S.; Poli, M.; Girardi, L.; Patassini, C.; Favero, F.; Cimadomo, D.; Vaiarelli, A.; Simon, C.; Rienzi, L. F.; Ubaldi, F. M. | 2021 | Human Reproduction | 36 | 7 | 2050-2061 | https://dx.doi.org/10.1093/humrep/deab087 | Outcomes are unclear or not relevant |
| Blastocysts from abnormally-fertilized zygotes can be euploid/diploid and are reproductively competent: Live-births from preimplantation genetic diagnosis of aneuploidy/polyploidy in embryos with abnormal pronuclear morphology | Capalbo, A.; Treff, N.; Cimadomo, D.; Tao, X.; Ferrero, S.; Vaiarelli, A.; Colamaria, S.; Giuliani, M.; Sapienza, F.; Ubaldi, F. M.; Scott, R.; Rienzi, L. | 2017 | Human Reproduction | 32 | SUPPL. 1 | i243 | 10.1093/humrep/32.Supplement_1.1 | Outcomes are unclear or not relevant |
| FISH reanalysis of inner cell mass and trophectoderm samples of previously array-CGH screened blastocysts shows high accuracy of diagnosis and no major diagnostic impact of mosaicism at the blastocyst stage | Capalbo, A.; Wright, G.; Elliott, T.; Ubaldi, F. M.; Rienzi, L.; Nagy, Z. P. | 2013 | Human Reproduction | 28 | 8 | 2298-2307 | http://dx.doi.org/10.1093/humrep/det245 | Use of FISH |
| Fish reanalysis of inner cell mass and trophecto-derm samples of previously array-CGH screened blastocysts reveals high accuracy of diagnosis and no sign of mosaicism or preferential allocation | Capalbo, A.; Wright, G.; Themaat, L.; Elliott, T.; Rienzi, L.; Nagy, Z. P. | 2011 | Fertility and Sterility | 96 | 3(SUPPL.) | S22 | http://dx.doi.org/10.1016/j.fertnstert.2011.07.094 | Duplicate |
| Comparison of array comparative genomic hybridization and quantitative real-time PCR-based aneuploidy screening of blastocyst biopsies | Capalbo, Antonio; Treff, Nathan R.; Cimadomo, Danilo; Tao, Xin; Upham, Kathleen; Ubaldi, Filippo Maria; Rienzi, Laura; Scott, Richard T., Jr. | 2015 | European Journal of Human Genetics | 23 | 7 | 901-906 | https://dx.doi.org/10.1038/ejhg.2014.222 | Wrong study design |
| New algorithmfor a precise determination of the level of mosaicism in Preimplantation Genetic Screening (PGS) with nextgeneration sequencing (NGS) | Castejon Fernandez, N.; Amoros, D.; Gonzalez-Reig, S.; Blanca, H.; Penacho, V.; Galan, F.; Alcaraz, L. A. | 2017 | Human Reproduction | 32 | SUPPL. 1 | i428-i429 | 10.1093/humrep/32.Supplement_1.1 | Inadequate information |
| Mosaicism within the day 3 embryo previously inferred as aneuploid from the first polar body | Cater, E.; Lynch, C.; Jenner, L.; Berrisford, K.; Campbell, A.; Keown, N.; Rouse, H.; Craig, A.; Fishel, S. | 2012 | Human Reproduction | 27 | SUPPL. 2 |  | 10.1093/humrep/27.s2.87 | Wrong study design |
| Predictive value of mural and pole trophectoderm samples of the chromosomal content of the inner cell mass (ICM) using array CGH technology | Cater, E.; Lynch, C.; Jenner, L.; Berrisford, K.; Campbell, A.; Thornton, S.; Fishel, S. | 2012 | Human Fertility | 15 | SUPPL. 1 | 7 |  | Inadequate information |
| Oocyte vitrification is a valid strategy to accumulate gametes for preimplantation genetic diagnosis for aneuploidy using next generation sequencing | Chamayou, S.; Sicali, M.; Alecci, C.; Ragolia, C.; Lombardo, D.; Storaci, G.; Romano, S.; Liprino, A.; Cardea, A.; Guglielmino, A. | 2016 | Human Reproduction | 31 | SUPPL. 1 | i399 | 10.1093/humrep/31.Supplement_1.1 | Inadequate information |
| 30. Live births following day 7 blastocyst transfer after preimplantation genetic testing for aneuploidy (PGT-A) | Chan, C. W.; Lim, Y. X.; Lim, M. W.; Lee, C. S. S.; Tan, C. S. | 2019 | Reproductive BioMedicine Online | 39 | SUPPL. 1 | e45 | 10.1016/j.rbmo.2019.04.083 | Outcomes are unclear or not relevant |
| Single-cell DNA sequencing reveals a high incidence of chromosomal abnormalities in human blastocysts | Chavli, E.A.; Klaasen, S.J.; Van Opstal, D.; Laven, J.S.; Kops G.; Baart, E.B. | 2024 | The Journal of Clinical Investigation | 134 | 6 | 1-11 | 10.1172/JCI174483 | Wrong study design |
| Pre-implantation genetic diagnosis: A preliminary report of 2 years of experience | Chen, C. K.; Wu, Y. C.; Shen, G. Y.; Lin, C. Y.; Wang, M. L.; Lee, M. W.; Huang, H. Y.; Wang, H. S.; Soong, Y. K. | 2004 | Chang Gung Medical Journal | 27 | 10 | 726-733 |  | Use of FISH |
| Segmental aneuploidy in PGT-A has the potential with euploidy in inner cell mass | Cheng, E.H.; Shih, H.H.; Lin, P.Y.; Lee, T.H.; Huang, C.C.; Lee, M.S. | 2022 | Fertility and Sterility | 118 | 4(SUPPL.) | E363 |  | Ina |
| Reanalysis of day 5 embryos following preimplantation genetic screening (PGS) for chromosomal abnormalities by fluorescence in situ hybridization on a single blastomere among different indication groups | Christopikou, D.; Tsorva, E.; Garantzioti, A.; Argyrou, M.; Moschopoulou, M.; Karagianni, A.; Mavrou, A.; Thornhill, A.; Griffin, D.; Davies, S.; Mastrominas, M. | 2010 | Reproductive BioMedicine Online | 20 | SUPPL. 1 | S17 |  | Use of FISH |
| Polar body analysis by array comparative genomic hybridization accurately predicts aneuploidies of maternal meiotic origin in cleavage stage embryos of women of advanced maternal age | Christopikou, Dimitra; Tsorva, Erika; Economou, Konstantinos; Shelley, Piran; Davies, Stephen; Mastrominas, Minas; Handyside, Alan H. | 2013 | Human Reproduction | 28 | 5 | 1426-1434 | https://dx.doi.org/10.1093/humrep/det053 | Wrong study design |
| Positive clinical outcome after preimplantation genetic screening (PGS) using newly developed whole genome array CGH platform after trophectoderm biopsy | Chung, M. K.; Kim, J. W.; Lee, J. H.; Jeong, H. J.; Kim, M. H.; Ryu, M. J.; Park, S. J.; Kang, H. Y.; Lee, H. S. | 2013 | Human Reproduction | 28 | SUPPL. 1 | i299 | 10.1093/humrep/det220 | Outcomes are unclear or not relevant |
| Quantitative polymerase chain reaction (qPCR)-based patterns consistent with mosaicism/partial aneuploidies(PA) indicate blastocysts with slightly lower reproductive competence: Preliminary results from a non-selection study | Cimadomo, D.; Patassini, C.; Giancani, A.; Scepi, E.; Dovere, L.; Romano, S.; Orlando, G.; Rienzi, L.; Ubaldi, F. M.; Capalbo, A. | 2017 | Human Reproduction | 32 | SUPPL. 1 | i429 | 10.1093/humrep/32.Supplement_1.1 | Outcomes are unclear or not relevant |
| The application of bioinformatics to genetic testing for the detection of human aneuploidy and genotyping | Cinnioglu, C. | 2012 | Reproductive BioMedicine Online | 24 | SUPPL. 2 | S37 | 10.1016/S1472-6483%2812%2960203-X | Inadequate information |
| New methods reveal the true incidence of DNA contamination of PGT-A samples for the first time and avoid errors that could result in serious misdiagnoses | Clark, G.; Babariya, D.; Del Canto Cano, A.; Fernandeze Marcos, E.; Parnell, L; Kilbee, M. Ceban, V.; Infantes, B.; Coudereau, C.; Spath, K.; Wells, D. | 2023 | Human Reproduction | 38 | SUPPL. 1 | i171 |  | Wrong comparator |
| Preimplantation genetic diagnosis at blastocyst stage by array comparative genomic hybridization. Error rate determination | Colls, P.; Coates, A.; Peters, A.; Acacio, B.; Roche, M.; Otani, T. | 2013 | Fertility and Sterility | 100 | 3(SUPPL.) | S196 | 10.1016/j.fertnstert.2013.07.1342 | Use of FISH |
| Reanalysis of monosomic embryos at the blastocyst stage following day 3 biopsy and array comparative genomic hybridization (aCGH) | Colls, P.; Ghadir, S.; Danzer, H.; Surrey, M.; Hill, D.; Munne, S. | 2011 | Fertility and Sterility | 95 | 4(SUPPL.) | S21 | 10.1016/j.fertnstert.2011.01.089 | Use of FISH |
| ESHRE PGT Consortium data collection XVI-XVIII: cycles from 2013 to 2015 | Coonen, E.; van Montfoort, A.; Carvalho, F.; Kokkali, G.; Moutou, C.; Rubio, C.; De Rycke, M.; Goossens, V. | 2020 | Human Reproduction Open | 2020 | 4 | hoaa043 | 10.1093/hropen/hoaa043 | Inadequate information |
| A retrospective study of preimplantation embryos diagnosed with monosomy by fluorescence in situ hybridization (FISH) | Cooper, M. L.; Darilek, S.; Wun, W. S.; Angus, S. C.; Mensing, D. E.; Pursley, A. N.; Dunn, R. C.; Grunert, G. M.; Cheung, S. W. | 2006 | Cytogenetic and Genome Research | 114 | 3-4 | 359-366 | https://doi.org/10.1159/000094226 | Use of FISH |
| FIRST REGISTERED PILOT TRIAL TO VALIDATE THE SAFETY AND EFFECTIVENESS OF MATERNAL SPINDLE TRANSFER TO OVERCOME INFERTILITY ASSOCIATED WITH POOR OOCYTE QUALITY | Costa-Borges, N.; Nikitos, E.; Spath, K.; Rink, K.; Kostaras, K.; Zervomanolakis, I.; Kontopoulos, G.; Polyzos, P.; Grigorakis, S.; Prokopakis, T.; Vasilopoulos, Y.; Vlahos, N.; de Ziegler, D.; Wells, D.; Psathas, P.; Calderon, G. | 2020 | Fertility and Sterility | 114 | 3(SUPPL.) | e71-e72 | 10.1016/j.fertnstert.2020.08.220 | Wrong study design |
| Copy number variation sequencing for preimplantation genetic diagnosis of chromosome abnormalities | Cram, D.; Wang, L.; Yao, Y. | 2014 | Prenatal Diagnosis | 34 | SUPPL. 1 | 72 | 10.1002/pd.4425 | Outcomes are unclear or not relevant |
| ESHRE PGD Consortium data collection XIII: cycles from January to December 2010 with pregnancy follow-up to October 2011 | De Rycke, M.; Belva, F.; Goossens, V.; Moutou, C.; SenGupta, S. B.; Traeger-Synodinos, J.; Coonen, E. | 2015 | Human Reproduction | 30 | 8 | 1763-1789 | 10.1093/humrep/dev122 | Inadequate information |
| ESHRE PGD Consortium data collection XIV-XV: cycles from January 2011 to December 2012 with pregnancy follow-up to October 2013 | De Rycke, M.; Goossens, V.; Kokkali, G.; Meijer-Hoogeveen, M.; Coonen, E.; Moutou, C. | 2017 | Human Reproduction | 32 | 10 | 1974-1994 | 10.1093/humrep/dex265 | Inadequate information |
| Accuracy of FISH analysis in predicting chromosomal status in patients undergoing preimplantation genetic diagnosis | DeUgarte, C. M.; Li, M.; Surrey, M.; Danzer, H.; Hill, D.; DeCherney, A. H. | 2008 | Fertility and Sterility | 90 | 4 | 1049-1054 | http://dx.doi.org/10.1016/j.fertnstert.2007.07.1337 | Use of FISH |
| Preimplantation genetic testing for human blastocysts with potential parental contamination using a quantitative parental contamination test (qPCT): an evidence-based study | Dong, Y.; Liu, D.; Zou, Y.; Wan, C.; Chen, C.; Dong, M.; Huang, Y.; Huang, C.; Weng, H.; Zhu, X.; Wang, F.; Jiao, S.; Liu, N.; Lu, S.; Zhang, X.; Liu, F. | 2023 | Reproductive Biomedicine Online | 46 | 1 | 69-79 | 10.1016/j.rbmo.2022.08.103 | Wrong study design |
| The black box of early pregnancy loss. Report of the 5th symposium of the Wim Schellenkens Foundation | Dorr, P. J. | 2004 | Nederlands Tijdschrift voor Obstetrie en Gynaecologie | 117 | 9 | 282-285 |  | Not English or French |
| Evaluation of preimplantation genetic aneuploidy screening cases at a reference genetics center: 10 years' experience | Durmaz, B.; Karaca, E.; Tavmergen Goker, E. N.; Tavmergen, E.; Sahin, G.; Akdogan, A.; Yasar, B. P.; Gunduz, C.; Ozkinay, R. | 2016 | Genetic Counseling | 27 | 4 | 461-470 |  | Duplicate |
| Evaluation of preimplantation genetic aneuploidy screening cases at a reference i genetics center: 10 years' experience | Durmaz, B.; Karaca, E.; Tavmergen Goker, E. N.; Tavmergen, E.; Sahn, G.; Akdogan, A.; Yasar, B. R.; Gunduz, C.; Ozkinay, F. | 2016 | Genetic Counseling | 27 | 4 | 461-470 |  | Use of FISH |
| Why Day 3 biopsy mosaicism is blamed for the lack of success of PGD-A? | Eibes, P.; Francis, E.; Chaturvedi, R.; Nawaz, M.; Shyju, S.; Thulasidas, D.; Hellani, A. | 2019 | Reproductive BioMedicine Online | 38 | SUPPL. 1 | e47 | http://dx.doi.org/10.1016/j.rbmo.2019.03.076 | Wrong study design |
| Enhancing embryos of the desired sex in couples undergoing PGT-A while minimizing embryo wastage | Elias, R.; Cheung, S.; Xie, P.; Rosenwaks, Z.; Palermo, G. D. | 2023 | Human Reproduction | 38 | SUPPL.1 | i206 | 10.1093/humrep/dead093.408 | Wrong study design |
| Comprehensive comparison of trophectoderm (TE) and inner cell mass (ICM) by next generation sequencing (NGS) | Elkhatib, I.; Lawrenz, B.; Linan, A.; Arnanz, A.; Bayram, A.; Fatemi, H. | 2018 | Human Reproduction | 33 | SUPPL. 1 | i410 | 10.1093/humrep/33.Supplement_1.1 | Duplicate |
| Comparison of the Validity of Preimplantation Genetic Diagnosis for Embryo Chromosomal Anomalies by Fluorescence In Situ Hybridization on One or Two Blastomeres | Emiliani, S.; Gonzalez-Merino, E.; Englert, Y.; Abramowicz, M. | 2004 | Genetic Testing | 8 | 1 | 69-72 | http://dx.doi.org/10.1089/109065704323016058 | Wrong study design |
| Successful preimplantation genetic aneuploidy screening in Turkish patients | Ercelen, N.; Turtar, E.; Gultomruk, M.; Comert, H.; Coskun, H.; Mercan, R.; Nuhoglu, A. | 2011 | Genetics and Molecular Research | 10 | 4 | 4093-103 | 10.4238/2011.November.17.6 | Use of FISH |
| Chromosome screening using culture medium of embryos fertilised in vitro: a pilot clinical study | Fang, R.; Yang, W.; Zhao, X.; Xiong, F.; Guo, C.; Xiao, J.; Chen, L.; Song, X.; Wang, H.; Chen, J.; Xiao, X.; Yao, B.; Cai, L.-Y. | 2019 | Journal of Translational Medicine | 17 | 73 |  | 10.1186/s12967-019-1827-1 | Wrong study design |
| Detection of embryo mosaicism with NGS | Farrell, M.; Grkovic, S.; Stockton, J.; McArthur, S. | 2019 | Twin Research and Human Genetics | 21 | 5 | 408 | 10.1017/thg.2018.51 | Inadequate information |
| A novel algorithm for determining the level of mosaicism in preimplantation genetic screening (PGS) with next-generation sequencing (NGS) | Fernandez, N. C.; Amoros, D.; Gonzalez-Reig, S.; Blanca, H.; Penacho, V.; Galan, F.; Alcaraz, L. A. | 2018 | Reproductive BioMedicine Online | 36 | SUPPL. 1 | e16 |  | Outcomes are unclear or not relevant |
| Validation and application of next-generation (NGS)-based protocol for 24-chromosome aneuploidy screening of embryos | Fiorentino, F. | 2014 | Chromosome Research | 22 | 4 | 593-594 | http://dx.doi.org/10.1007/s10577-014-9437-5 | Wrong study design |
| PREGNANCY OUTCOME OF TRANSFERRED EMBRYOS WITH MOSAICISM AND SEGMENTAL VARIATIONS | Fiorentino, F. | 2019 | Reproductive BioMedicine Online | 39 | SUPPL. 1 | e9-e10 | 10.1016/j.rbmo.2019.04.029 | Outcomes are unclear or not relevant |
| Clinical outcome derived after tranfer of embryos with chromosomal mosaicism | Fiorentino, F.; Biricick, A.; Bono, S.; Greco, E.; Minasi, M. G.; Ruberti, A.; Spinella, F. | 2016 | Human Reproduction | 31 | SUPPL. 1 | i13 | 10.1093/humrep/31.Supplement_1.1 | Outcomes are unclear or not relevant |
| Application of next-generation sequencing technology for comprehensive aneuploidy screening of blastocysts in clinical preimplantation genetic screening cycles | Fiorentino, F.; Bono, S.; Biricik, A.; Nuccitelli, A.; Cotroneo, E.; Cottone, G.; Kokocinski, F.; Michel, C. E.; Minasi, M. G.; Greco, E. | 2014 | Human Reproduction | 29 | 12 | 2802-2813 | http://dx.doi.org/10.1093/humrep/deu277 | Comparator is not relevant |
| Polar body (PB) based aneuploidy screening is significantly less predictive of the reproductive potential of embryos than embryo biopsy based techniques | Forman, E. J.; Salvaggio, C. N.; Garnsey, H. M.; Treff, N. R.; Scott Jr, R. T. | 2013 | Fertility and Sterility | 100 | 3(SUPPL.) | S199-S200 | http://dx.doi.org/10.1016/j.fertnstert.2013.07.1353 | Wrong study design |
| Single embryo transfer with comprehensive chromosome screening results in improved ongoing pregnancy rates and decreased miscarriage rates | Forman, E. J.; Tao, X.; Ferry, K. M.; Taylor, D.; Treff, N. R.; Scott, R. T., Jr. | 2012 | Human Reproduction | 27 | 4 | 1217-1222 | 10.1093/humrep/des020 | Outcomes are unclear or not relevant |
| Analysis of implantation and ongoing pregnancy rates following the transfer of mosaic diploid-aneuploid blastocysts | Fragouli, E.; Alfarawati, S.; Spath, K.; Babariya, D.; Tarozzi, N.; Borini, A.; Wells, D. | 2017 | Human Genetics | 136 | 7 | 805-819 | https://dx.doi.org/10.1007/s00439-017-1797-4 | Outcomes are unclear or not relevant |
| Cytogenetic analysis of human blastocysts with the use of FISH, CGH and aCGH: scientific data and technical evaluation | Fragouli, Elpida; Alfarawati, Samer; Daphnis, Danny D.; Goodall, N.-Neka; Mania, Anastasia; Griffiths, Tracey; Gordon, Anthony; Wells, Dagan | 2011 | Human Reproduction | 26 | 2 | 480-490 | 10.1093/humrep/deq344 | Use of FISH |
| Clinical error rates of next generation sequencing (NGS) compared to array comparative genomic hybridization (ACGH) in euploid blastocysts | Friedenthal, J.; Maxwell, S.M.; Tiegs, A.W.; Besser, A.; McCaffrey, C.; Munne, S.; Noyes, N.; Grifo, J. | 2017 | Fertility and Sterility | 108 | 3 | e297 | 10.1016/j.fertnstert.2017.07.878 | Duplicate |
| The Paris experience in preimplantation genetic diagnosis: Evaluation after the first births | Frydman, N.; Romana, S.; Ray, P.; Hamamah, S.; Tachdjian, G.; Marcadet-Fredet, S.; Munnich, A.; Vekemans, M.; Frydman, R. | 2005 | Annales d'Endocrinologie | 66 | 3 | 294-301 |  | Wrong patient population |
| Pre-implantation genetic screening using fluorescence in situ hybridization in couples of Indian ethnicity: Is there a scope? | Gada Saxena, S.; Desai, K.; Shewale, L.; Ranjan, P. | 2014 | Journal of Human Reproductive Sciences | 7 | 1 | 25-29 | 10.4103/0974-1208.130812 | Use of FISH |
| Optimized NGS based protocol for the detection of small duplications/deletions in preimplantation embryos from carriers of, balanced translocations and inversions | Garcia Pascual, C. M.; Navarro-Sanchez, L.; Martinez-Merino, L.; Rodrigo, L.; Simon, C.; Rubio, C. | 2018 | Human Reproduction | 33 | SUPPL. 1 | i409 | http://dx.doi.org/10.1093/humrep/33.Supplement_1.1 | Duplicate |
| 11. HIGH CONSISTENCY IN EMBRYO ANEUPLOIDY TESTING OF UNIFORM, MOSAIC AND SEGMENTAL ANEUPLOIDIES WITH THE APPLICATION OF A VALIDATED ALGORITHM | Garcia Pascual, C.; Navarro Sanchez, L.; Navarro Gaya, R.; Rodrigo Vivo, L.; Garcia Herrero, S.; Campos Galindo, I.; Peinado Cervera, V.; Jimenez Almazan, J.; Simon Valles, C.; Rubio Lluesa, C. | 2019 | Reproductive BioMedicine Online | 39 | SUPPL. 1 | e20-e21 | 10.1016/j.rbmo.2019.04.046 | Inadequate information |
| Concurrent pre-implantation genetic testing for single gene disorders and aneuploidy screening from a single trophectoderm (TE) biopsy using targeted next generation sequencing (NGS) without whole genome amplification (WGA) | Garnsey, H.; Jalas, C.; Zhan, Y.; Vega, C.; Jobanputra, V.; Scott, R. T.; Tao, X. | 2019 | Fertility and Sterility | 112 | 3(SUPPL.) | e32-e33 | 10.1016/j.fertnstert.2019.07.216 | Wrong study design |
| A small trophectoderm biopsy sample is sufficient to detect most mosaicisms after analysis with high resolution next generation sequencing (NGS) | Garrisi, G. J.; Walmsley, R.; Bauckman, K.; Mendola, R.; Colls, P.; Munne, S. | 2016 | Human Reproduction | 31 | SUPPL. 1 | i406-i407 | 10.1093/humrep/31.Supplement_1.1 | Inadequate information |
| The timing of onset of mosaicism in human blastocysts impacts the utility of next generation sequencing (NGS) | Garrisi, G.; Walmsley, R. H.; Bauckman, K.; Dela Cerna, C.; Colls, P.; Munne, S. | 2016 | Fertility and Sterility | 106 | 3(SUPPL.) | e147 |  | Inadequate information |
| The beneficial effects of preimplantation genetic diagnosis for aneuploidy support extensive clinical application | Gianaroli, L.; Magli, M. C.; Ferraretti, A. P.; Tabanelli, C.; Trengia, V.; Farfalli, V.; Cavallini, G. | 2005 | Reproductive BioMedicine Online | 10 | 5 | 633-40 | 10.1016/s1472-6483(10)61671-9 | Use of FISH |
| Preimplantation genetic testing for aneuploidy in patients with partial X monosomy using their own oocytes: is this a suitable indication? | Giles, J. Meseguer, M.; Mercader, A.; Rubio, C.; Alegre, L.; Vidal, C.; Trabalon, M.; Bosch, E. | 2020 | Fertility and Sterility | 114 | 2 | 346-353 | 10.1016/j.fertnstert.2020.04.003 | Outcomes are unclear or not relevant |
| Embryos diagnosed as putative mosaic by the PGTSEQ PGT-A platform have a similar sustained implantation rate as those negative for putative mosaicism: a blinded non-selection study | Gill, P.; Zhan, Y.; Whitehead, C. V.; Tao, X.; Werner, M. D.; Molinaro, T.; Scott, R. T.; Jalas, C. | 2022 | Fertility and Sterility | 118 | 4(SUPPL.) | E29 | 10.1016/j.fertnstert.2022.08.101 | Wrong outcomes |
| Segmental aneuploidies show mosaic pattern reducing predictive value compared to high whole chromosome aneuploidies representativeness | Girardi, L.; Romanelli, V.; Fabiani, M.; Cimadomo, D.; Rienzi, L.; Ubaldi, F. M.; Serdarogullari, M.; Coban, O.; Findikli, N.; Boynukalin, K.; Bahceci, M.; Patassini, C.; Poli, M.; Lluesa, C. R.; Simon, C.; Capalbo, A. | 2019 | Reproductive BioMedicine Online | 39 | SUPPL. 1 | e18-e19 | http://dx.doi.org/10.1016/j.rbmo.2019.04.043 | Duplicate |
| Preimplantation genetic screening (PGS) appears unable to correctly determine ploidy of embryos from a single trophectoderm biopsy (TEB) | Gleicher, N.; Vidali, A.; Kushnir, V. A.; Barad, D. H.; Hudson, C.; Wang, Q.; Zhang, L.; Albertini, D. | 2016 | Fertility and Sterility | 106 | 3(SUPPL.) | e371 |  | Inadequate information |
| [Preimplantation genetic diagnosis (PGD): the Erasme Hospital experience] | Gonzalez-Merino, E.; Emiliani, S.; Pichon, B.; Parma, J.; Vannin, A. S.; Delbaere, A.; Vassart, G.; Abramowicz, M.; Englert, Y. | 2008 | Le Diagnostic Genetique Preimplantatoire (DPI): L'experience de L'Hopital Erasme. | 29 | 6 | 527-534 |  | Inadequate information |
| Evaluation of next generation sequencing (NGS) based comprehensive chromosome screening (CCS) sensitivity to mosaicism | Goodrich, D.; Tao, X.; Bohrer, C.; Bedard, J.; Landis, J. N.; Scott, R. T.; Treff, N. R. | 2015 | Fertility and Sterility | 104 | 3(SUPPL.) | e280-e281 |  | Duplicate |
| An evidence-based scoring system for prioritizing mosaic aneuploid embryos following preimplantation genetic screening | Grati, F. R.; Gallazzi, G.; Branca, L.; Maggi, F.; Simoni, G.; Yaron, Y. | 2018 | Reproductive BioMedicine Online | 36 | 4 | 442-449 | https://dx.doi.org/10.1016/j.rbmo.2018.01.005 | Wrong study design |
| Human blastocysts derived from monopronuclear zygotes: a biological model for the study of ploidy, euploidy, topography and heteroparental inheritance | Grau, N.; Soler, N.; Gonzalez-Picazo, A.; Vendrell, X.; Escriba, M. J.; Gamiz, P. | 2019 | Fertility and Sterility | 112 | 3(SUPPL.) | e46-e47 | 10.1016/j.fertnstert.2019.07.250 | Inadequate information |
| Successful blastocyst biopsy and Preimplantation Genetic Screening after embryo cryopreservation and extended culture: Analysis on 213 frozen-thawed supernumerary embryos, previously cryopreserved without biopsy | Greco, E.; Minasi, M. G.; Ruberti, A.; Casciani, V.; Scarselli, F.; Colasante, A.; Cursio, E.; Lobascio, M.; Greco, A.; Greco, P.; Caragia, A.; Varricchio, M. T.; Biricik, A.; Spinella, F.; Fiorentino, F. | 2016 | Human Reproduction | 31 | SUPPL. 1 | i210 | 10.1093/humrep/31.Supplement_1.1 | Outcomes are unclear or not relevant |
| The mosaic embryo: what it means for the doctor and the patient | Greco, E.; Greco, P.F.; Listorti, I.; Ronsini, C.; Cucinelli, F.; Biricik, A.; Viotti, M.; Meschino, N.; Spinella, F. | 2024 | Minerva Obstetrics and Gynecology | 76 | 1 | 89-101 | 10.23736/S2724-606X.23.05281-8 | Wrong study design |
| Direct detection of FMR1 CGG repeats causative for fragile x syndrome coupled with 24-chromosome aneuploidy screening in single cells | Handschuh, K.; Zhang, C.; Qin, X.; Rosenwaks, Z.; Xu, K. | 2014 | Fertility and Sterility | 102 | 3(SUPPL.) | e185 | 10.1016/j.fertnstert.2014.07.624 | Inadequate information |
| Re-analysis of 166 embryos not transferred after PGS with advanced reproductive maternal age as indication | Hanson, C.; Hardarson, T.; Lundin, K.; Bergh, C.; Hillensjo, T.; Stevic, J.; Westin, C.; Selleskog, U.; Rogberg, L.; Wikland, M. | 2009 | Human Reproduction | 24 | 11 | 2960-2964 | 10.1093/humrep/dep264 | Use of FISH |
| Detection of genetic mosaicism during preimplantation genetic diagnosis (PGD) | Harasim, T.; Roesemann, M.; Heinrich, U.; Wagner, A.; Schiller, J.; Rozek, M.; Kotanidou, A.; Montag, M.; Klein, H. G.; Rost, I. | 2013 | Reproductive BioMedicine Online | 26 | SUPPL. 1 | S21 | 10.1016/S1472-6483%2813%2960052-8 | Outcomes are unclear or not relevant |
| NIPT RESULTS IN PREGNANCIES RESULTING FROM EUPLOID EMBRYO TRANSFERS | Harjee, R.; Nakhuda, G. S.; Jing, C. | 2020 | Fertility and Sterility | 114 | 3(SUPPL.) | e156-e157 | 10.1016/j.fertnstert.2020.08.450 | Wrong study design |
| Data from the ESHRE PGD consortium | Harper, J.; Goossens, V.; Harton, G. | 2010 | Human Reproduction | 25 | SUPPL. 1 | i17-i18 | 10.1093/humrep/de.25.s1.9 | Inadequate information |
| The ESHRE PGD Consortium: 10 years of data collection | Harper, J.C.; Wilton, L.; Traeger-Synodinos, J.; Goossens, V.; Moutou, C.; SenGupta, S.B.; Pehlivan Budak, T.; Renwick, P.; De Rycke, M.; Geraedts, J.P.M.; Harton, G. | 2012 | Human Reproduction Update | 18 | 3 | 234-247 | 10.1093/humupd/dmr052 | Inadequate information |
| Data from the ESHRE PGD Consortium | Harton, G.; Traeger-Syndinos, J.; Goossens, V. | 2011 | Human Reproduction | 26 | SUPPL. 1 | i17-i18 | 10.1093/humrep/26.s1.9 | Inadequate information |
| Data from the ESHRE PGD consortium | Harton, G.; Traeger-Syndinos, J.; Goossens, V. | 2012 | Human Reproduction | 27 | SUPPL. 2 | i58 | 10.1093/humrep/27.s2.40 | Inadequate information |
| The identification of chromosome deletions in trophectoderm biopsies is significantly representative of the entire blastocyst | Henry, L.; Mann, R. S.; McReynolds, S.; McCubbin, N.; Jarvis, T.; Schoolcraft, W. B.; Katz-Jaffe, M. G. | 2019 | Fertility and Sterility | 112 | 3(SUPPL.) | E229 |  | Duplicate |
| The chances of obtaining a euploid embryo and subsequent live birth remain consistent with national age-based rates after an in vitro fertilization cycle that produced only aneuploid embryos | Herlihy, N.S.; Klimczak, A. M.; Cheung, J. K. W.; Seli, E.; Scott, R. T., Jr. | 2022 | Fertility and Sterility | 118 | 3 | 484-491 | https://dx.doi.org/10.1016/j.fertnstert.2022.05.026 | Outcomes are unclear or not relevant |
| Report on first 105 patients undergoing preimplantation genetic diagnosis for genetic disorders together with 24 chromosome aneuploidy screening | Hill, M.; Wemmer, N.; Potter, D.; Keller, J.; Gemelos, G.; Rabinowitz, M. | 2012 | Human Reproduction | 27 | SUPPL. 2 |  | 10.1093/humrep/27.s2.87 | Inadequate information |
| Discordance rates between day 3 and day 6 chromosome results and the predictive value of time-lapse morphokinetics | Ho, J. R.; Arrach, N.; Salem, W.; Ingles, S. A.; Bendikson, K.; Chung, K.; Paulson, R.; Ahmady, A. | 2016 | Fertility and Sterility | 106 | 3(SUPPL.) | e157 |  | Wrong study design |
| Comparison of SNP-array and next generation sequencing in preimplantation genetic testing | Hong, Z.; Yueyun, L.; Jinhui, S.; Caizhu, W.; Xin, Z.; Lifang, L.; Sheng, H.; Qingming, Q.; Peng, H. | 2023 | Chinese Journal of Reproduction and Contraception | 43 | 10 | 1018-1025 | 10.3760/cma.j.cn101441-20220908-00390 | Not in English |
| National reference materials of preimplantation genetic aneuploid screening based on next generation sequencing | Huang, J.; Liu, P.; Chen, F.; Jiang, H.; Qiu, Y.; Chen, D. Y.; Xia, J.; Xie, L.; Zhu, Z.; Shi, Q. Y. | 2017 | Human Reproduction | 32 | SUPPL. 1 | i414 | 10.1093/humrep/32.Supplement_1.1 | Outcomes are unclear or not relevant |
| Validation of a next-generation sequencing-based protocol for 24-chromosome aneuploidy screening of blastocysts | Huang, J.; Yan, L.; Lu, S.; Zhao, N.; Xie, X. S.; Qiao, J. | 2016 | Fertility and Sterility | 105 | 6 | 1532-1536 | http://dx.doi.org/10.1016/j.fertnstert.2016.01.040 | Wrong study design |
| High efficacy of non-invasive chromosome screening using spent culture medium for preimplantation genetic testing of human embryos | Huang, L.; Bogale, B.; Lu, S.; Xie, X. S.; Racowsky, C. | 2017 | Fertility and Sterility | 108 | 3(SUPPL.) | e277-e278 |  | Duplicate |
| Pre-implantation genetic diagnosis as a key parameter for the success of in vitro fertilization | Ismayilova, M. K. | 2018 | Azerbaijan Medical Journal | 2018 | 4 | 23-28 |  | Not English or French |
| Highly abnormal cleavage divisions in preimplantation embryos from translocation carriers | Iwarsson, E.; Malmgren, H.; Inzunza, J.; Ahrlund-Richter, L.; Sjoblom, P.; Rosenlund, B.; Fridstrom, M.; Hovatta, O.; Nordenskjold, M.; Blennow, E. | 2000 | Prenatal Diagnosis | 20 | 13 | 1038-1047 |  | Wrong study design |
| Combined PGD and PGS by NGS on the same biopsy using a single index | Jasper, M.; Brockman, M.; Hodgson, B.; Warren, K. | 2018 | Reproductive BioMedicine Online | 36 | SUPPL. 1 | e16-e17 |  | Inadequate information |
| Comprehensive analysis of karyotypic mosaicism between trophectoderm and inner cell mass | Johnson, D.S.; Cinnioglu, C.; Ross, R.; Filby, A.; Gemelos, G.; Hill, M.; Ryan, A.; Smotrich, D.; Rabinowitz, M.; Murray, M.J. | 2010 | Molecular Human Reproduction | 16 | 12 | 944-949 | 10.1093/molehr/gaq062 | Outcomes are unclear or not relevant |
| Preimplantation genetic diagnosis (PGD) for inherited disorders using single nucleotide polymorphism (SNP) arrays: Clinical outcomes of 100 cycles with transferable embryos | Jordan, A.; Prates, R.; Goodall, N.; Armenti, E.; Tecson, V.; Munne, S.; Konstantinidis, M.; Jaroudi, S. | 2015 | Human Reproduction | 30 | SUPPL. 1 | i396-i397 | 10.1093/humrep/30.Supplement-1.1 | Inadequate information |
| Clinical analysis of preimplantation genetic diagnosis of 36 cases of male infertility with Y chromosome abnormality | Jun, W.; Yaning, L.; Yefei, M.; Li, L.; Sai, L.; Shun, Z.; Jianlei, H.; Qinli, H.; Xiaohong, W. | 2012 | Chinese Journal of Andrology | 26 | 8 | 45212 | 10.3969/j.issn.1008-0848.2012.08.003 | Unable to access full text |
| Reliable detection of segmental aneuploidy identified by next generation sequencing (NGS) | Juneau, C. R.; Scott, K.; Neal, S.; Morin, S. J.; Zhan, Y.; Zimmerman, R. S.; Treff, N.; Franasiak, J. M.; Scott, R. T. | 2016 | Fertility and Sterility | 106 | 3(SUPPL.) | e377 |  | Inadequate information |
| The results of aneuploidy screening in 276 couples undergoing assisted reproductive techniques | Kahraman, S.; Benkhalifa, M.; Donmez, E.; Biricik, A.; Sertyel, S.; Findikli, N.; Berkil, H. | 2004 | Prenatal Diagnosis | 24 | 4 | 307-311 |  | Outcomes are unclear or not relevant |
| Preimplantation genetic screening: who benefits? | Kang, H-J.; Melnick, A.P.; Stewart, J.D.; Xu, K.; Rosenwaks, Z. | 2016 | Fertility and Sterility | 106 | 3 | 597-602 | 10.1016/j.fertnstert.2016.04.027 | Outcomes are unclear or not relevant |
| Usefulness of combined NGS and QF-PCR analysis for product of conception karyotyping | Kato, T.; Miyai, S.; Suzuki, H.; Murase, Y.; Ota, S.; Yamauchi, H.; Ammae, M.; Nakano, T.; Nakaoka, Y.; Inoue, T.; Morimoto, Y.; Fukuda, A.; Utsunomiya, T.; Nishizawa, H.; Kurahashi, H. | 2022 | Reproductive Medicine and Biology | 21 | 1 | E12449 | 10.1002/rmb2.12449 | Wrong study design |
| Clinical application of comprehensive chromosome screening and beyond | Katz-Jaffe, M. | 2011 | Biology of Reproduction | 85 | SUPPL.1 | 111 | https://doi.org/10.1093/biolreprod/85.s1.111 | Inadequate information |
| Chromosome analysis in embryos from young patients with previous parity | Kilani, Z.; Magli, Mc; Qaddomi, E.; Ferraretti, Ap; Shaban, M.; Crippa, A.; Haj Hassan, L.; Shenfield, F.; Gianaroli, L. | 2014 | Reproductive BioMedicine Online | 29 | 3 | 333-339 | https://dx.doi.org/10.1016/j.rbmo.2014.04.018 | Wrong study design |
| Confirmation rate of whole chromosome calls on a targeted next-generation sequencing platform are highly consistent with initial trophectoderm biopsy | Kim, J. G.; Tao, X.; Zhan, Y.; Cheng, M.; Zhao, T.; Guo, V.; Hanson, B. M.; Scott, R. T.; Jalas, C. | 2020 | Fertility and Sterility | 114 | 3(SUPPL.) | e414-e415 | 10.1016/j.fertnstert.2020.08.1209 | Inadequate information |
| The concordance rates of an initial trophectoderm biopsy with the rest of the embryo using PGTseq, a targeted next-generation sequencing platform for preimplantation genetic testing-aneuploidy | Kim, J.; Tao, X.; Cheng, M.; Steward, A.; Guo, V.; Zhan, Y.; Scott, R. T., Jr.; Jalas, C. | 2022 | Fertility and Sterility | 117 | 2 | 315-323 | https://dx.doi.org/10.1016/j.fertnstert.2021.10.011 | Comparator is not relevant |
| Non-invasive prenatal testing has altered positive predictive value following transfer of a euploid blastocyst | Klimczak, A. M.; Whitehead, C. V.; Neal, S. A.; Tiegs, A. W.; Osman, E. K.; Hanson, B. M.; Kim, J. G.; Werner, M. D.; Franasiak, J. M.; Scott, R. T. | 2019 | Fertility and Sterility | 112 | 3(SUPPL.) | e18 | http://dx.doi.org/10.1016/j.fertnstert.2019.07.180 | Duplicate |
| Preimplantation genetic diagnosis outcomes and meiotic segregation analysis of robertsonian translocation carriers | Ko, D. S.; Cho, J. W.; Lee, H. S.; Kim, J. Y.; Kang, I. S.; Yang, K. M.; Lim, C. K. | 2013 | Fertility and Sterility | 99 | 5 | 1369-1376 | 10.1016/j.fertnstert.2012.12.010 | Wrong patient population |
| Genetic outcomes of conception in men with elevated sperm aneuploidy | Kohn, T. P.; Pastuszak, A. W.; Cherches, M. F.; Pascoe, K. F.; Shah, S.; Lamb, D. J.; Lipshultz, L. I. | 2017 | Journal of Urology | 197 | 4(SUPPL.) | e81 |  | Inadequate information |
| A novel tool for the assessment of IVF embryos | Konstantinidis, M.; Alfarawati, S.; Hurd, D.; Wells, D. | 2011 | Human Reproduction | 26 | SUPPL. 1 | i24-i25 | 10.1093/humrep/26.s1.17 | Outcomes are unclear or not relevant |
| Multiplex ligation-dependent probe amplification (MLPA) as a stand-alone test for rapid aneuploidy detection in amniotic fluid cells | Kooper, A. J.; Faas, B. H.; Kater-Baats, E.; Feuth, T.; Janssen, J. C.; van der Burgt, I.; Lotgering, F. K.; van Kessel, A. G.; Smits, A. P. | 2008 | Prenatal Diagnosis | 28 | 11 | 1004-1010 | 10.1002/pd.2111 | Wrong patient population |
| Is there any difference in chromosome abnormalities at human embryos derived via ICSI with or without PolScope? | Korkmaz, C.; Bahce, M.; Baykal, B.; Akyol, M.; Duru, N. K.; Baser, I. | 2013 | Reproductive BioMedicine Online | 26 | SUPPL. 1 | S32 | 10.1016/S1472-6483%2813%2960076-0 | Inadequate information |
| Validation of a next generation sequencing-based preimplantation genetic screening assay for the calling of triploidy and uniparental isodisomy | Kosheleva, K.; Velenich, A.; Sartain, C.; Faulkner, N.; Robinson, K.; Zhu, M.; Porreca, G.; Umbarger, M. | 2018 | Fertility and Sterility | 109 | 3 | e54 |  | Inadequate information |
| First experiences with preimplantation genetic screening of chromosomal aberrations using oligonucleotide-based array comparative genomic hybridization | Kuglik, P.; Smetana, J.; Nemcova, D.; Vallova, V.; Mikulasova, A.; Gaillyova, R.; Hubinka, V.; Koudelka, M. | 2015 | Casopis Lekaru Ceskych | 154 | 3 | 127-131 |  | Not English or French |
| Chromosomal abnormalities in a series of 6,733 human oocytes in preimplantation diagnosis for age-related aneuploidies | Kuliev, A.; Cieslak, J.; Ilkevitch, Y.; Verlinsky, Y. | 2003 | Reproductive BioMedicine Online | 6 | 1 | 19968 |  | Wrong study design |
| Polar body based PGD for genetic and chromosomal disorders | Kuliev, A.; Zlatopolsky, Z.; Kirillova, I.; Ilkevitch, Y.; Cieslak Janzen, J. | 2012 | Reproductive BioMedicine Online | 24 | SUPPL. 2 | S31 | 10.1016/S1472-6483%2812%2960187-4 | Wrong study design |
| Degree of mosaicism in trophectoderm does not predict pregnancy potential: a corrected analysis of pregnancy outcomes following transfer of mosaic embryos | Kushnir, V. A.; Darmon, S. K.; Barad, D. H.; Gleicher, N. | 2018 | Reproductive Biology and Endocrinology | 16 | 1 | 6 | https://dx.doi.org/10.1186/s12958-018-0322-5 | Wrong study design |
| Non-invasive preimplantation genetic screening of human blastocysts | Kuznyetsov, V.; Madjunkova, S.; Antes, R.; Abramov, R.; Motamedi, G.; Ibarrientos, Z.; Librach, C. L. | 2017 | Fertility and Sterility | 108 | 3(SUPPL.) | e277 |  | Inadequate information |
| Live births after transfer of rebiopsy and revitrification of blastocyst that had ''no diagnosis'' following trophectoderm biopsy | Lee, H.; McCulloh, D. H.; Olivares, R.; Goldstein-Tufaro, A.; McCaffrey, C.; Grifo, J. | 2016 | Fertility and Sterility | 106 | 3(SUPPL.) | e164 |  | Wrong study design |
| Preimplantation genetic testing is not a preferred recommendation for patients with X chromosome abnormalities | Li, C.; Dang, Y.; Li, J.; Li, H.; Zhu, Y.; Qin, Y. | 2021 | Human Reproduction | 36 | 9 | 2612-2621 | https://dx.doi.org/10.1093/humrep/deab177 | Outcomes are unclear or not relevant |
| Development and validation of a pacgenomics-agilent microarray system for preimplantation genetic screening (PGS) in patients undergoing in vitro fertilization (IVF) | Li, M.; Jin, H.; Snyder, R.; Zhou, J.; Liu, L. | 2013 | Fertility and Sterility | 100 | 3(SUPPL.) | S198 | http://dx.doi.org/10.1016/j.fertnstert.2013.07.1347 | Inadequate information |
| Chromosome copy number variations among embryonic blastomeres revealed by single cell sequencing | Li, W.; Cram, D.; Jianguang, Z.; Xiaohong, W.; Yuanqing, Y. | 2013 | Reproductive BioMedicine Online | 26 | SUPPL. 1 | S13-S14 | 10.1016/S1472-6483%2813%2960035-8 | Wrong study design |
| A healthy live birth after successful preimplantation genetic diagnosis for carriers of complex chromosome rearrangements | Lim, Chun Kyu; Cho, Jae Won; Kim, Jin Yeong; Kang, Inn Soo; Shim, Sung Han; Jun, Jin Hyun | 2008 | Fertility and Sterility | 90 | 5 | 1680-1684 |  | Wrong patient population |
| Feasibility study of repeated fluorescent in-situ hybridization in the same human blastomeres for preimplantation genetic diagnosis | Liu, J.; Tsai, Y. L.; Zheng, X. Z.; Yazigi, R. A.; Baramki, T. A.; Compton, G.; Katz, E. | 1998 | Molecular Human Reproduction | 4 | 10 | 972-977 |  | Wrong study design |
| Assessment of clinical application of preimplantation genetic screening on cryopreserved human blastocysts | Liu, M.; Su, Y.; Wang, W.-H. | 2016 | Reproductive Biology and Endocrinology | 14 | 16 |  | 10.1186/s12958-016-0155-z | Outcomes are unclear or not relevant |
| Enhanced sensitivity provided by single cell SNP array analysis allows detection of clinically significant villous mosaicism not detected by conventional cytogenetics | Lonczak, A.; Tao, X.; Garnsey, H. M.; Treff, N. R.; Scott Jr, R. T. | 2013 | Fertility and Sterility | 100 | 3(SUPPL.) | S325 | http://dx.doi.org/10.1016/j.fertnstert.2013.07.941 | Inadequate information |
| Noninvasive prenatal testing (NIPT) after euploid embryo transfer shows high concordance with PGT-A results and low NIPT predictive values | Madjunkov, M.; Abramov, R.; Glass, K. B.; Baratz, A. Y.; Sharma, P. A.; Madjunkova, S.; Librach, C. L. | 2023 | Fertility and Sterility | 120 | 4(SUPPL.) | e50 | 10.1016/j.fertnstert.2023.08.165 | Wrong study design |
| Evidence-based management of mosaic embryos-a single centre experience from prospective transfer and birth outcomes of 565 mosaic embryos | Madjunkov, M.; Balakier, H.; Abramov, R.; Chen, S.; Logan, N.; Baratz, A.; Glass, K.; Sharma, P.; Madjunkova, S.; Librach, C. | 2023 | Human Reproduction | 39 | SUPPL.1 | i120 | 10.1093/humrep/dead093.237 | Wrong outcomes |
| Chromosome mosaicism in day 3 aneuploid embryos that develop to morphologically normal blastocysts in vitro | Magli, M. C.; Jones, G. M.; Gras, L.; Gianaroli, L.; Korman, I.; Trounson, A. O. | 2000 | Human Reproduction | 15 | 8 | 1781-1786 |  | Use of FISH |
| Double locus analysis of chromosome 21 for preimplantation genetic diagnosis of aneuploidy | Magli, M. C.; Sandalinas, M.; Escudero, T.; Morrison, L.; Ferraretti, A. P.; Gianaroli, L.; Munne, S. | 2001 | Prenatal Diagnosis | 21 | 12 | 1080-1085 |  | Use of FISH |
| Detection of aneuploidy by array comparative genomic hybridization using cell lines to mimic a mosaic trophectoderm biopsy | Mamas, T.; Gordon, A.; Brown, A.; Harper, J.; Sengupta, S. | 2012 | Fertility and Sterility | 97 | 4 | 943-947 | https://dx.doi.org/10.1016/j.fertnstert.2011.12.048 | Outcomes are unclear or not relevant |
| Variable aneuploidy mechanisms in embryos from couples with poor reproductive histories undergoing preimplantation genetic screening | Mantzouratou, A.; Mania, A.; Fragouli, E.; Xanthopoulou, L.; Tashkandi, S.; Fordham, K.; Ranieri, D. M.; Doshi, A.; Nuttall, S.; Harper, J. C.; Serhal, P.; Delhanty, J. D. | 2007 | Human Reproduction | 22 | 7 | 1844-1853 | 10.1093/humrep/dem102 | Use of FISH |
| A novel next generation sequencing (NGS)-based comprehensive chromosome screening (CCS) platform that provides accurate copy number and genotyping in parallel from a single trophectoderm biopsy | Marin, D.; Zimmerman, R.; Jalas, C.; Tao, X.; Zhan, Y.; Scott, R.; Treff, N. | 2017 | Human Reproduction | 32 | SUPPL. 1 | i16-i17 | 10.1093/humrep/32.Supplement_1.1 | Duplicate |
| Preimplantation genetic testing for aneuploidies does not increase success rates in fresh oocyte donation cycles: a paired cohort study | Martello, C. L.; Kulmann, M. I. R.; Donatti, L. M.; Bos-Mikich, A.; Frantz, N. | 2021 | Journal of Assisted Reproduction and Genetics | 38 | 11 | 2909-2914 | https://dx.doi.org/10.1007/s10815-021-02339-2 | Outcomes are unclear or not relevant |
| Technique to ascertain the chromosomal content of the inner cell mass without compromising embryo development | Matt, D.; Purcell, S.; Jones, A.; Williams, C.; Wilkerson, B.; Collier, J.; Locksley, R. | 2017 | Fertility and Sterility | 108 | 3(SUPPL.) | e58 |  | Inadequate information |
| Effect of the male factor on the clinical outcome of intracytoplasmic sperm injection combined with preimplantation aneuploidy testing: observational longitudinal cohort study of 1,219 consecutive cycles | Mazzilli, R.; Cimadomo, D.; Vaiarelli, A.; Capalbo, A.; Dovere, L.; Alviggi, E.; Dusi, L.; Foresta, C.; Lombardo, F.; Lenzi, A.; Tournaye, H.; Alviggi, C.; Rienzi, L.; Ubaldi, F. M. | 2017 | Fertility and Sterility | 108 | 6 | 961-972.e3 | https://dx.doi.org/10.1016/j.fertnstert.2017.08.033 | Comparator is not relevant |
| Rebiopsy of blastocysts reveals that next generation sequencing provides excellent clinical accuracy despite minor discordances | McCulloh, D. H.; Sachdev, N.; McCaffrey, C.; Grifo, J. A. | 2019 | Fertility and Sterility | 112 | 3(SUPPL.) | e231-e232 | 10.1016/j.fertnstert.2019.07.716 | Duplicate |
| PATERNAL SEGMENTAL CHROMOSOME CONFUSION | McReynolds, S.; Henry, L.; McCubbin, N.; Mann, R. S.; Tucci, R.; McCormick, S.; Schoolcraft, W. B.; Katz-Jaffe, M. G. | 2020 | Fertility and Sterility | 114 | 3(SUPPL.) | e358 | 10.1016/j.fertnstert.2020.08.1068 | Inadequate information |
| Oligonucleotide based array CGH test performance for detection of chromosomal imbalances in single cells | Melotte, C.; Dimitriadou, E.; Vermeesch, J. R. | 2014 | Chromosome Research | 22 | 4 | 630 | 10.1007/s10577-014-9437-5 | Inadequate information |
| VALIDATION OF PREIMPLANTATION GENETIC TESTS FOR ANEUPLOIDY WITH CELL-FREE DNA FROM SPENT CULTURE MEDIA (SCM): CONCORDANCE ASSESSMENT AND IMPLICATION | Meng, L.; Yin, B.; Zhang, C. | 2020 | Fertility and Sterility | 114 | 3(SUPPL.) | e420-e421 | http://dx.doi.org/10.1016/j.fertnstert.2020.08.1224 | Inadequate information |
| Genetic diseases and aneuploidies can be detected with a single blastocyst biopsy: a successful clinical approach | Minasi, M. G.; Fiorentino, F.; Ruberti, A.; Biricik, A.; Cursio, E.; Cotroneo, E.; Varricchio, M. T.; Surdo, M.; Spinella, F.; Greco, E. | 2017 | Human Reproduction | 32 | 8 | 1770-1777 | 10.1093/humrep/dex215 | Outcomes are unclear or not relevant |
| Sex discordance in frozen embryo transfer (FET) cycles: a marker for quality assurance (QA) | Minis, E.; Jiang, V. S.; Fitz, V. W.; Dimitriadis, I.; Souter, I.; Bormann, C. L. | 2023 | Fertility and Sterility | 120 | 4(SUPPL.) | e220 | 10.1016/j.fertnstert.2023.08.630 | Wrong comparator |
| False positive rate of an arrayCGH platform for single-cell preimplantation genetic screening and subsequent clinical application on day-3 | Mir, P.; Rodrigo, L.; Mercader, A.; Buendia, P.; Mateu, E.; Milan-Sanchez, M.; Peinado, V.; Pellicer, A.; Remohi, J.; Simon, C.; Rubio, C. | 2013 | Journal of Assisted Reproduction and Genetics | 30 | 1 | 143-149 | https://dx.doi.org/10.1007/s10815-012-9918-4 | Use of FISH |
| A novel method for single cell dissection and sequencing of human blastocysts | Molinari, E.; Escudero, T.; Sawarkar, S. S.; Munne, S.; Patrizio, P. | 2017 | Fertility and Sterility | 108 | 3(SUPPL.) | e276 |  | Wrong study design |
| ESHRE PGD Consortium data collection XII: cycles from January to December 2009 with pregnancy follow-up to October 2010 | Moutou, C.; Goossens, V.; Coonen, E.; De Rycke, M.; Kokkali, G.; Renwick, P.; SenGupta, S. B.; Vesela, K.; Traeger-Synodinos, J. | 2014 | Human Reproduction | 29 | 5 | 880-903 | 10.1093/humrep/deu012 | Inadequate information |
| Detailed investigation into the cytogenetic constitution and pregnancy outcome of replacing mosaic blastocysts detected with the use of high-resolution next-generation sequencing | Munne, S.; Blazek, J.; Large, M.; Martinez-Ortiz, P.A.; Nisson, H.; Liu, E.; Tarozzi, N.; Borini, A.; Becker, A.; Zhang, J.; Maxwell, S.; Grifo, J.; Babariya, D.; Wells, D.; Fragouli, E. | 2017 | Fertility and Sterility | 107 | 6 | 62-71.e8 | 10.1016/j.fertnstert.2017.05.002 | Wrong study design |
| Detailed investigation into the cytogenetic constitution and pregnancy outcome of replacing mosaic blastocysts detected with the use of high-resolution next-generation sequencing | Munne, S.; Blazek, J.; Large, M.; Martinez-Ortiz, P.A.; Nisson, H.; Liu, E.; Tarozzi, N.; Borini, A.; Becker, A.; Zhang, J.; Maxwell, S.; Grifo, J.; Babariya, D.; Wells, D.; Fragouli, E. | 2017 | Fertility and Sterility | 108 | 1 | 62-71.e8 | https://dx.doi.org/10.1016/j.fertnstert.2017.05.002 | Duplicate |
| Scoring criteria for preimplantation genetic diagnosis of numerical abnormalities for chromosomes X, Y, 13, 16, 18 and 21 | Munne, S.; Marquez, C.; Magli, C.; Morton, P.; Morrison, L. | 1998 | Molecular Human Reproduction | 4 | 9 | 863-870 |  | Use of FISH |
| Self-correction of chromosomally abnormal embryos in culture and implications for stem cell production | Munne, S.; Velilla, E.; Colls, P.; Bermudez, M. G.; Vemuri, M. C.; Steuerwald, N.; Garrisi, J.; Cohen, J. | 2005 | Fertility and Sterility | 84 | 5 | 1328-1334 | 10.1016/j.fertnstert.2005.06.025 | Wrong study design |
| Simultaneous enumeration of chromosomes 13, 18, 21, X, and Y in interphase cells for preimplantation genetic diagnosis of aneuploidy | Munne, S.; Weier, H. U. | 1996 | Cytogenetics and Cell Genetics | 75 | 4 | 263-270 |  | Use of FISH |
| To transfer or not to transfer: the dilemma of mosaic embryos - a narrative review | Munoz, E.; Bronet, F.; Lledo, B.; Palacios-Verdu, G.; Martinez-Rocca, L.; Altmae, S.; Pla, J.; Representing the Special Interest Group in Reproductive Genetics of the Spanish Society of, Fertility | 2024 | Reproductive BioMedicine Online | 48 | 3 | 103664 | 10.1016/j.rbmo.2023.103664 | Wrong study design |
| High concordance of the embryonic cell-free DNA with the inner cell mass: impact of blastocyst quality, patient age and mode of fertilization | Navarro Sanchez, L.; Ocali, O.; Garcia Pascual, C. M.; Mamede Andrade, G.; Castello Salom, D.; Lai, F.; Gross Dutra, C.; Rubio, C.; Simon, C.; Frantz, N.; Sakkas, D. | 2022 | Human Reproduction | 37 | SUPPL.1 | i448-i449 | 10.1093/humrep/deac106.P-551 | Inadequate information |
| Consistency of embryo cell-free DNA results with paired trophectoderm biopsies and whole day-6 blastocysts in different culture conditions | Navarro-Sanchez, L.; Rodrigo, L.; Ceschin, I. I.; Campos-Galindo, I.; Picasso, A. P.; Lopez De Carvajal, L. M.; Rivadeneira, A.; Castello, D.; Al-Asmar, N.; Simon, C.; Lluesa, C. R. | 2023 | Fertility and Sterility | 120 | 4(SUPPL.) | e221-e222 | 10.1016/j.fertnstert.2023.08.633 | Wrong comparator |
| Comparison of various aneuploidy types among two trophectoderm biopsies and a rest of the embryo | Navratil, R.; Horak, J.; Hornak, M.; Kubicek, D.; Oracova, E.; Janickova, N.; Vesely, J.; Vesela, K. | 2019 | Reproductive BioMedicine Online | 39 | SUPPL. 1 | e20 | http://dx.doi.org/10.1016/j.rbmo.2019.04.045 | Duplicate |
| Single nucleotide polymorphism (SNP) microarray preimplantation genetic screening (PGS): A comparison between abnormal day-3 blastomeres and corresponding inner cell mass and trophectoderm cells | Nguyen, D. K. H.; Ross, R.; Benner, A.; Zhao, Y.; Brezina, P.; Kearns, W. G. | 2010 | Fertility and Sterility | 94 | 4(SUPPL.) | S80 | 10.1016/j.fertnstert.2010.07.311 | Use of FISH |
| Embryo quality, but not the level and the type of mosaicism is the most important factor influencing pregnancy outcomes of single mosaic embryo transfers | Nhu Nguyen Thi Nhu, T. T.; Thi Cam, V. N.; Tran, T. H.; Thi Phuong, L. L.; Van, H. T.; Huu, Q. K. | 2022 | Fertility and Sterility | 118 | 4(SUPPL.) | e359 | 10.1016/j.fertnstert.2022.09.174 | Inadequate information |
| Accumulation of oocytes and/or embryos by vitrification: A new strategy for managing poor responder patients undergoing pre implantation diagnosis | Nijs, M.; Chatziparasidou, A.; Moisidou, M.; Chara, O.; Ioakeimidou, C.; Pappas, C.; Christoforidis, N. | 2014 | F1000Research | 2 | 240.v2 | 1-10 | 10.12688/f1000research.2-240.v2 | Outcomes are unclear or not relevant |
| PGD for translocations-increased risk for multiple chromosomal abnormalities | Okada, L.; Sanseverino, M. T.; Azambuja, R.; Tagliani-Ribeiro, A.; Badalotti, M.; Petracco, A. | 2013 | Fertility and Sterility | 100 | 3(SUPPL.) | S207 | 10.1016/j.fertnstert.2013.07.1380 | Inadequate information |
| A workflow for simultaneous DNA copy number and methylome analysis of ICM and TE cells from human blastocysts | Olcha, M.; Dong, X.; Feil, H.; Hao, X.; Lee, M.; Jindal, S.; Buyuk, E.; Vijg, J. | 2019 | Fertility and Sterility | 111 | 4(SUPPL.) | e5-e6 | 10.1016/j.fertnstert.2019.02.042 | Inadequate information |
| The reproducibility of trophectoderm biopsies - The chaos behind preimplantation genetic testing for aneuploidy | Orvieto, Raoul | 2020 | European Journal of Obstetrics, Gynecology, and Reproductive Biology | 254 |  | 57-58 | 10.1016/j.ejogrb.2020.07.052 | Wrong study design |
| 24. PREIMPLANTATION GENETIC TESTING FOR ANEUPLOIDIES WITH NGS ON MINISEQ. A RELIABLE ALTERNATIVE FOR SMALL LABORATORIES | Papaevripidou, I.; Alexandrou, A.; Constantinou, E.; Theodosiou, A.; Evangelidou, P.; Sismani, C. | 2019 | Reproductive BioMedicine Online | 39 | SUPPL. 1 | e41-e42 | http://dx.doi.org/10.1016/j.rbmo.2019.04.077 | Comparator is not relevant |
| Clinical significance of undiagnosed mosaicism in IVF embryos | Perry, E.; Beyer, C.; Willats, E.; Lin, J.; Low, L. Y.; Mullen, J.; Rombauts, L. | 2018 | Reproductive BioMedicine Online | 36 | SUPPL. 1 | e14 |  | Inadequate information |
| Pre-implantation genetic screening for discrimination of carrier and non-carrier reciprocal translocation with array-CGH and PGD | Pingsuthiwong, S.; Tiewsiri, K.; Jantapanon, T. K.; Narknava, Y.; Marshall, J. | 2018 | Reproductive BioMedicine Online | 36 | SUPPL. 1 | e38 |  | Wrong study design |
| A successful strategy for Preimplantation Genetic Diagnosis of beta-thalassemia and simultaneous detection of Down's syndrome using multiplex fluorescent PCR | Piyamongkol, W.; Vutyavanich, T.; Piyamongkol, S.; Wells, D.; Kunaviktikul, C.; Tongsong, T.; Chaovisitsaree, S.; Saetung, R.; Sanguansermsri, T. | 2006 | Journal of the Medical Association of Thailand = Chotmaihet thangphaet | 89 | 7 | 918-927 |  | Wrong study design |
| Reporting chromosomal mosaicism reduces the overall accuracy of preimplantation genetic testing for aneuploidies: results from the extended in vitro culture of 230 human embryos | Popovic, M.; Azpiroz, F.; Pujol, A.; Lledo, B.; Franco, J. M.; Aurell, R.; Urries, A.; Fernandez, C.; Menten, B.; Rodriguez, A. | 2023 | Human Reproduction | 38 | SUPPL.1 | I539-i540 | 10.1093/humrep/dead093.1061 | Inadequate information |
| Adapting next-generation DNA sequencing to detect aneuploidy | Porreca, G. J.; Gole, J.; Gore, A.; Umbarger, M. A. | 2014 | Fertility and Sterility | 102 | 3(SUPPL.) | e181-e182 | http://dx.doi.org/10.1016/j.fertnstert.2014.07.612 | Inadequate information |
| Targeted copy number analysis for preimplantation genetic screening | Porreca, G.; Gole, J.; Gore, A.; Boyden, E.; Slevin, M.; Maganzini, D.; Saunders, P.; Umbarger, M. | 2015 | Human Reproduction | 30 | SUPPL. 1 | i393-i394 | http://dx.doi.org/10.1093/humrep/30.Supplement-1.1 | Duplicate |
| IVF outcomes on patients Who underwent preimplantation genetic diagnosis (PGD) for inherited genetic disorders with concurrent 24 chromosome aneuploidy screening using single nucleotide polymorphism (SNP) microarrays | Potter, D.; Wemmer, N.; Merrion, K.; Hill, M.; Rabinowitz, M. | 2013 | Fertility and Sterility | 99 | 3(SUPPL.) | S26-S27 | 10.1016/j.fertnstert.2013.01.057 | Inadequate information |
| Results of array comparative genomic hybridization (ACGH) combined with multiplex pcr for preimplantation genetic diagnosis (PGD) of both single gene disorders and aneuploidy | Prates, R.; Kung, A.; Fischer, J. M.; Grifo, J.; Kaplan, B.; Escudero, T. | 2012 | Fertility and Sterility | 98 | 3(SUPPL.) | S137 | 10.1016/j.fertnstert.2012.07.506 | Inadequate information |
| Report on first 54 patients undergoing preimplantation genetic diagnosis for genetic disorders together with 24 chromosome aneuploidy screening using microarrays | Rabinowitz, M.; Hill, M.; Potter, D.; Wemmer, N.; Keller, J.; Gemelos, G. | 2011 | Fertility and Sterility | 96 | 3(SUPPL.) | S23 | 10.1016/j.fertnstert.2011.07.1144 | Inadequate information |
| Causes and estimated incidences of sex-chromosome misdiagnosis in preimplantation genetic diagnosis of aneuploidy | Ravichandran, K.; Guzman, L.; Escudero, T.; Zheng, X.; Colls, P.; Jordan, A.; Cohen, J.; Wells, D.; Munne, S. | 2016 | Reproductive BioMedicine Online | 33 | 5 | 550-559 | 10.1016/j.rbmo.2016.08.011 | Wrong study design |
| First systematic experience of 24-chromosome aneuploidy testing in PGD for HLA and single gene disorders | Rechitsky, S.; Verlinsky, O.; Kuliev, A. | 2013 | Fertility and Sterility | 100 | 3(SUPPL.) | S129-S130 | 10.1016/j.fertnstert.2013.07.1603 | Inadequate information |
| Regional and developmental characteristics of human embryo mosaicism revealed by single cell sequencing | Ren, Y.; Yan, Z.; Yang, M.; Keller, L.; Zhu, X.; Lian, Y.; Liu, Q.; Li, R.; Zhai, F.; Nie, Y.; Yan, L.; Smith, G.D.; Qiao, J. | 2022 | PLoS Genetics | 18 | 8 | e1010310 | 10.1371/journal.pgen.1010310 | Wrong study design |
| A deep dive into the human blastocyst: Gene based sex differences in human embryos and the assessment of aneuploidy by transcriptome analysis-progress and limitations | Resetkova, N.; Groff, A.; Sakkas, D.; Penzias, A.; Rinn, J. L.; Eggan, K. | 2017 | Fertility and Sterility | 108 | 3(SUPPL.) | e152 |  | Wrong study design |
| Embryo selection versus natural selection: how do outcomes of comprehensive chromosome screening of blastocysts compare with the analysis of products of conception from early pregnancy loss (dilation and curettage) among an assisted reproductive technology population? | Rodriguez-Purata, Jorge; Lee, Joseph; Whitehouse, Michael; Moschini, Rose Marie; Knopman, Jaime; Duke, Marlena; Sandler, Benjamin; Copperman, Alan | 2015 | Fertility and Sterility | 104 | 6 | 1460-1466 | <https://dx.doi.org/10.1016/j.fertnstert.2015.08.007> | Comparator is not relevant |
| Healthy baby after intrauterine transfer of monosomic embryos | Rubino, P.; Dearden, L.; Guan, L.; Ruiz De Assin, R.; Mazmanian, K.; Kolb, B. A.; Nelson, J.; Norian, J. M.; Wilcox, J.; Tan, T. | 2016 | Fertility and Sterility | 106 | 3(SUPPL.) | e160 |  | Outcomes are unclear or not relevant |
| In vitro fertilization with preimplantation genetic diagnosis for aneuploidies in advanced maternal age: a randomized, controlled study | Rubio, C.; Bellver, J.; Rodrigo, L.; Castillon, G.; Guillen, A.; Vidal, C.; Giles, J.; Ferrando, M.; Cabanillas, S.; Remohi­, J.; Pellicer, A.; Simon, Carlos | 2017 | Fertility and Sterility | 107 | 5 | 1122-1129 | 10.1016/j.fertnstert.2017.03.011 | Outcomes are unclear or not relevant |
| Reliability of next generation sequencing (NGS) diagnosis of aneuploidy or mosaicism by re-biopsy and repeat NGS of inner cell mass (ICM) and trophectoderm (TE) fromhuman embryos | Sachdev, N. M.; Kramer, Y. G.; Meyn, P.; McCulloh, D. H.; Grifo, J.; Keefe, D. L. | 2017 | Fertility and Sterility | 108 | 3(SUPPL.) | e87 |  | Duplicate |
| Validation of Next-Generation Sequencer for 24-Chromosome Aneuploidy Screening in Human Embryos | Sachdeva, K.; Discutido, R.; Albuz, F.; Almekosh, R.; Peramo, B. | 2017 | Genetic Testing and Molecular Biomarkers | 21 | 11 | 674-680 | http://dx.doi.org/10.1089/gtmb.2017.0108 | Wrong study design |
| The fate of the mosaic embryo: Chromosomal constitution and development of Day 4, 5 and 8 human embryos | Santos, M. A.; Teklenburg, G.; MacKlon, N. S.; Van Opstal, D.; Schuring-Blom, G. H.; Krijtenburg, P. J.; De Vreeden-Elbertse, J.; Fauser, B. C.; Baart, E. B. | 2010 | Human Reproduction | 25 | 8 | 1916-1926 | 10.1093/humrep/deq139 | Use of FISH |
| The impact of chromosomal mosaicism on early embryonic development | Santos, M. A.; Teklenburg, G.; Van Opstal, D.; Schuring-Blom, G. H.; Macklon, N. S.; Baart, E. B. | 2010 | Reproductive Sciences | 17 | SUPPL. 3 | 243A-244A | 10.1177/193371912010173s067 | Wrong study design |
| The role of preimplantation genetic testing for aneuploidy in a good prognosis IVF population across different age groups | Sarkar, P.; Jindal, S.; New, E. P.; Sprague, R. G.; Tanner, J.; Imudia, A. N. | 2021 | Systems Biology in Reproductive Medicine | 67 | 5 | 366-373 | https://dx.doi.org/10.1080/19396368.2021.1954725 | Outcomes are unclear or not relevant |
| Preimplantation diagnosis by fluorescence in situ hybridization using 13-, 16-, 18-, 21-, 22-, X-, and Y-chromosome probes | Sasabe, Y.; Katayama, K. P.; Nishimura, T.; Takahashi, A.; Asakura, H.; Winchester-Peden, K.; Wise, L.; Abe, Y.; Kubo, H.; Hirakawa, S. | 1999 | Journal of Assisted Reproduction and Genetics | 16 | 2 | 92-96 |  | Use of FISH |
| First clinical application of SNP microarray based 24 chromosome aneuploidy screening of human blastocysts | Schoolcraft, W. B.; Treff, N. R.; Ferry, K.; Stevens, J. M.; Katz-Jaffe, M. G.; Scott, R. T. | 2010 | Fertility and Sterility | 94 | 4(SUPPL.) | S23-S24 | http://dx.doi.org/10.1016/j.fertnstert.2010.07.090 | Outcomes are unclear or not relevant |
| Preimplantation diagnosis of aneuploidy using fluorescent in-situ hybridization: evaluation using a chromosome 18-specific probe | Schrurs, B. M.; Winston, R. M.; Handyside, A. H. | 1993 | Human Reproduction | 8 | 2 | 296-301 |  | Wrong study design |
| Single cell rapid testing technique for preimplantation genetic screening on the BGISEQ-50 | Shi, Q.; Chen, F.; Liu, P.; Chen, D.; Qiu, Y.; Xia, J.; Zhu, Z.; Xie, L. | 2017 | Human Reproduction | 32 | SUPPL. 1 | i421 | 10.1093/humrep/32.Supplement_1.1 | Inadequate information |
| Validation of the de-novo segmental (>16 megabase) loss detected by next generation sequencing in 24 blastocysts from preimplantation genetics diagnosis | Shuang, Z.; Dehua, C.; Pingyuan, X.; Qi, O.; Liang, H.; Bo, X.; Guangxiu, L.; Ge, L. | 2016 | Human Reproduction | 31 | SUPPL. 1 | i401 | 10.1093/humrep/31.Supplement_1.1 | Duplicate |
| Preimplantation genetic diagnosis of chromosome abnormalities: implications from the outcome for couples with chromosomal rearrangements | Simopoulou, M.; Harper, J. C.; Fragouli, E.; Mantzouratou, A.; Speyer, B. E.; Serhal, P.; Ranieri, D. M.; Doshi, A.; Henderson, J.; Rodeck, C. H.; Delhanty, J. D. A. | 2003 | Prenatal Diagnosis | 23 | 8 | 652-662 |  | Wrong patient population |
| Development and validation of an array-based nano-scale quantitative polymerase chain reaction technology for comprehensive chromosome screening of preimplantation embryos | Spath, K.; Alfarawati, S.; Fragouli, E.; Wells, D. | 2015 | Human Fertility | 18 | 4 |  | http://dx.doi.org/10.3109/14647273.2015.1060045 | Inadequate information |
| Clinical application of sequencing-based methods for parallel preimplantation genetic testing for mitochondrial DNA disease and aneuploidy | Spath, K.; Babariya, D.; Konstantinidis, M.; Lowndes, J.; Child, T.; Grifo, J.A.; Poulton, J.; Wells, D. | 2021 | Fertility and Sterility | 115 | 6 | 1521-1532 | https://dx.doi.org/10.1016/j.fertnstert.2021.01.026 | Outcomes are unclear or not relevant |
| Development, validation and first clinical application of a novel ultra-rapid comprehensive chromosome screening technique utilising an array based nano scale quantitative DNA amplification technology | Spath, K.; Kubikova, N.; Whitney, M.; Vaid, M.; Rozis, G.; Couchman, V.; Glynn, K.; Batha, S.; Alfarawati, S.; Fragouli, E.; Wells, D. | 2017 | Human Reproduction | 32 | SUPPL. 1 | i16-i17 | 10.1093/humrep/32.Supplement_1.1 | Inadequate information |
| The extent of chromosomal mosaicism influences the clinical outcome of in vitro fertilization treatments | Spinella, F.; Biricik, A.; Bono, S.; Minasi, M. G.; Cotroneo, E.; Baldi, M.; Cursio, E.; Diano, L.; Greco, E.; Fiorentino, F. | 2018 | Reproductive BioMedicine Online | 36 | SUPPL. 1 | e11 | 10.1016/j.rbmo.2017.10.027 | Duplicate |
| Next generation sequencing-based aneuploidy screening improves detection of low-level mosaicism in human embryos | Spinella, F.; Biricik, A.; Bono, S.; Nuccitelli, A.; Cotroneo, E.; Cottone, G.; Kokocinski, F.; Michel, C. E.; Fiorentino, F. | 2014 | Fertility and Sterility | 102 | 3(SUPPL.) | e94-e95 | http://dx.doi.org/10.1016/j.fertnstert.2014.07.322 | Wrong study design |
| The extent of chromosomal mosaicism influences the clinical outcome of in vitro fertilization treatments | Spinella, F.; Biricik, A.; Minasi, M.; Greco, E.; Fiorentino, F. | 2017 | Fertility and Sterility | 108 | 3(SUPPL.) | e272 | 10.1016/j.fertnstert.2017.07.809 | Duplicate |
| Preventing the discard of potentially transferable embryos by next generation sequencing-based preimplantation genetic screening | Spinella, F.; Bono, S.; Biricik, A.; Barberi, M.; Nuccitelli, A.; Kokocinski, F.; Michel, C.; Minasi, M. G.; Greco, E.; Fiorentino, F. | 2015 | Human Reproduction | 30 | SUPPL. 1 | i399 | http://dx.doi.org/10.1093/humrep/30.Supplement-1.1 | Outcomes are unclear or not relevant |
| Clinical experience of sex determination by fluorescent in-situ hybridization for preimplantation genetic diagnosis | Staessen, C.; Van Assche, E.; Joris, H.; Bonduelle, M.; Vandervorst, M.; Liebaers, I.; Van Steirteghem, A. | 1999 | Molecular Human Reproduction | 5 | 4 | 382-389 |  | Use of FISH |
| Single-cell analysis of human embryos reveals diverse patterns of aneuploidy and mosaicism | Starosti, M. R.; Sosin, O. A.; McCo, R. C. | 2020 | Genome Research | 30 | 6 | 814-826 | 10.1101/gr.262774.120 | Wrong study design |
| First experiences with PGD after trophectoderm biopsy at Kinderwunsch Centrum Munich (KCM), Germany | Suttner, R.; Shakeshaft, D.; Koehler, U.; Schon, U.; Harasim, Th; Wagner, A.; Holinski-Feder, E.; Rost, I.; Wurfel, W. | 2012 | Reproductive BioMedicine Online | 24 | SUPPL. 2 | S48 | 10.1016/S1472-6483%2812%2960225-9 | Inadequate information |
| How to choose prenatal testing options for pregnant women after preimplantation genetic screening (PGT-A): Genetic counseling challenges | Tamura, C.; Arakawa, H.; Fujita, S.; Nakamura, Y. | 2020 | Prenatal Diagnosis | 40 | SUPPL. 1 | 9 | 10.1002/pd.5624 | Wrong study design |
| Chromosome copy analysis by single-cell comparative genomic hybridization technique based on primer extension preamplification and degenerate oligonucleotide primed-PCR | Tan, K.; Di, Y. F.; Cheng, D. H.; Xu, F.; Lu, G. X.; Tan, Y. Q. | 2010 | Chinese Journal of Medical Genetics | 27 | 4 | 387-392 | 10.3760/cma.j.issn.1003-9406.2010.04.006 | Not English or French |
| 21. REPEATED VITRIFICATION AND WARMING PROCEDURES HAS NO DETRIMENTAL EFFECT ON BLASTOCYST SURVIVAL AND PREGNANCY OUTCOME | Tan, S. H.; Lee, C. S. S.; Lim, Y. X.; Lim, M. W.; Chan, C. W. | 2019 | Reproductive BioMedicine Online | 39 | SUPPL. 1 | e39-e40 | 10.1016/j.rbmo.2019.04.074 | Inadequate information |
| Array comparative genomic hybridisation for use in clinical preimplantation genetic screening | Thornhill, A.; Taylor, J.; Gordon, T.; Griffin, D.; Affara, N.; Handyside, A. | 2009 | Human Fertility | 12 | 4 | 230 | 10.3109/14647270903271913 | Inadequate information |
| Does preimplantation genetic testing for aneuploidy (PGT-A) harm embryos? no-a multi-center, prospective, blinded, non-selection study evaluating the predictive value of an aneuploid diagnosis and impact of biopsy | Tiegs, A. W.; Tao, X.; Whitehead, C. V.; Neal, S. A.; Osman, E. K.; Kim, J. G.; Hanson, B. M.; Seli, E.; Patounakis, G.; Gutmann, J.; Castelbaum, A. J.; Scott, R. T. | 2019 | Fertility and Sterility | 112 | 3(SUPPL.) | e31 | http://dx.doi.org/10.1016/j.fertnstert.2019.07.213 | Duplicate |
| TRANSFER OUTCOMES OF EMBRYOS WITH PREIMPLANTATION GENETIC TESTING FOR ANEUPLOIDY (PGT-A) DIAGNOSES OF UNDETERMINED REPRODUCTIVE POTENTIAL: RESULTS FROM A PROSPECTIVE, BLINDED, MULTI-CENTER NON-SELECTION STUDY | Tiegs, A. W.; Tao, X.; Zhan, Y.; Whitehead, C. V.; Hanson, B. M.; Kim, J. G.; Osman, E. K.; Seli, E.; Patounakis, G.; Gutmann, J.; Castelbaum, A. J.; Kim, T.; Jalas, C.; Scott, R. T. | 2020 | Fertility and Sterility | 114 | 3(SUPPL.) | e32 | http://dx.doi.org/10.1016/j.fertnstert.2020.08.115 | Outcomes are unclear or not relevant |
| A MULTI-CENTER, PROSPECTIVE, BLINDED, NON-SELECTION STUDY EVALUATING THE PREDICTIVE VALUE (PV) OF AN ANEUPLOID DIAGNOSIS WITH PGT-A AND THE IMPACT OF BIOPSY | Tiegs, A. W.; Tao, X.; Zhan, Y.; Whitehead, C. V.; Seli, E.; Patounakis, G.; Gutmann, J.; Castelbaum, A. J.; Kim, T.; Jalas, C.; Scott, R. T. | 2020 | Fertility and Sterility | 114 | 3(SUPPL.) | e30 | http://dx.doi.org/10.1016/j.fertnstert.2020.08.111 | Outcomes are unclear or not relevant |
| The potential use of blastocoel fluid (BF) from expanded blastocysts as a less invasive form of embryo biopsy for preimplantation genetic testing | Tobler, K. J.; Zhao, Y.; Ross, R.; Benner, A. T.; Xu, X.; Du, L.; Broman, K.; Thrift, K.; Brezina, P. R.; Kearns, W. G. | 2014 | Fertility and Sterility | 102 | 3(SUPPL.) | e183-e184 | http://dx.doi.org/10.1016/j.fertnstert.2014.07.618 | Duplicate |
| Next-Generation Sequencing (NGS)-Based Preimplantation Genetic Testing for Aneuploidy (PGT-A) of Trophectoderm Biopsy for Recurrent Implantation Failure (RIF) Patients: a Retrospective Study | Tong, J.; Niu, Y.; Wan, A.; Zhang, T. | 2021 | Reproductive Sciences | 28 | 7 | 1923-1929 | https://dx.doi.org/10.1007/s43032-021-00519-0 | Outcomes are unclear or not relevant |
| Reanalysis of human blastocysts with different molecular genetic screening platforms reveals significant discordance in ploidy status | Tortoriello, D. V.; Dayal, M.; Beyhan, Z.; Yakut, T.; Keskintepe, L. | 2016 | Journal of Assisted Reproduction and Genetics | 33 | 11 | 1467-1471 | http://dx.doi.org/10.1007/s10815-016-0766-5 | Wrong study design |
| Data from the ESHRE PGD consortium | Traeger-Synodinos, J.; Coonen, E. C.; De Rycke, M.; Moutou, C.; SenGupta, S.; Goossens, V. | 2014 | Human Reproduction | 29 | SUPPL. 1 | i18-19 | 10.1093/humrep/29.Supplement_1.1 | Inadequate information |
| Data from the ESHRE PGD Consortium | Traeger-Synodinos, J.; Coonen, E.; Goossens, V. | 2013 | Human Reproduction | 28 | SUPPL. 1 | i18-19 | 10.1093/humrep/det162 | Inadequate information |
| Mosaicism detection by QPCR versus NGS based CCS | Treff, N. R.; Goodrich, D.; Tao, X.; Zhan, Y.; Scott, R. T. | 2016 | Fertility and Sterility | 106 | 3(SUPPL.) | e153-e154 |  | Inadequate information |
| Duplication and deletion detection limits of veriseq PGS | Treff, N. R.; Lonczak, A.; Tao, X.; Jalas, C.; Gabriele, D. A.; Scott, R. T. | 2015 | Fertility and Sterility | 104 | 3(SUPPL.) | e275 |  | Inadequate information |
| Characterizing the nature of meiotic errors: PSSC is the principal meiotic error in human oocytes and may correct during the 2ND meiotic division and result in the birth of a healthy infant | Treff, N. R.; Su, J.; Tao, X.; Katz-Jaffe, M.; Schoolcraft, W.; Scott, R. | 2009 | Fertility and Sterility | 92 | 3(SUPPL.) | S25 |  | Inadequate information |
| Development and validation of a next-generation sequencing (NGS)-based 24-chromosome aneuploidy screening system | Treff, N. R.; Tao, X.; Taylor, D.; Hong, K. H.; Forman, E. J.; Scott Jr, R. T. | 2013 | Fertility and Sterility | 100 | 3(SUPPL.) | S82 | 10.1016/j.fertnstert.2013.07.1955 | Inadequate information |
| First IVF babies born after rapid 24 chromosome embryo aneuploidy screening and fresh embryo transfer | Treff, N.; Su, J.; Tao, X.; Miller, K.; Scott, R. | 2009 | Fertility and Sterility | 92 | 3(SUPPL.) | S49 |  | Wrong study design |
| Four hour 24 chromosome aneuploidy screening using high throughput PCR SNP allele ratio analyses | Treff, N.; Tao, X.; Su, J.; Taylor, D.; Miller, K.; Scott, R. | 2009 | Fertility and Sterility | 92 | 3(SUPPL.) | S49-S50 |  | Inadequate information |
| First babies born after preimplantation microarray analysis (PMA) for chromosome translocation carriers | Treff; Levy, B.; Kasabwala, N. R. K.; Su, J.; Garnsey, H.; Scott, R. T. | 2010 | Fertility and Sterility | 94 | 4(SUPPL.) | S79 | 10.1016/j.fertnstert.2010.07.307 | Use of FISH |
| 9. ASSESSMENT OF ANEUPLOIDY AND MOSAICISM CONCORDANCE BETWEEN DIFFERENT TROPHECTODERM BIOPSY SITES AND THE INNER CELL MASS EVALUATED WITH NEXT-GENERATION SEQUENCING | Tufekci, M. A.; Cetinkaya, M.; Yapan, C. C.; Colakoglu, Y. K.; Yelke, H.; Cetinkaya, C. P.; Kahraman, S. | 2019 | Reproductive BioMedicine Online | 39 | SUPPL. 1 | e19-e20 | 10.1016/j.rbmo.2019.04.044 | Inadequate information |
| Preimplantation genetic diagnosis for aneuploidy testing in women older than 44 years: a multicenter experience | Ubaldi, F. M. ; Cimadomo, D.; Capalbo, A.; Vaiarelli, A.; Buffo, L.; Trabucco, E.; Ferrero, S.; Albani, E.; Rienzi, L.; Levi Setti, P. E. | 2017 | Fertility and Sterility | 107 | 5 | 1173-1180 | 10.1016/j.fertnstert.2017.03.007 | Outcomes are unclear or not relevant |
| Accurate detection of segmental aneuploidy in preimplantation genetic screening using targeted next-generation DNA sequencing | Umbarger, M. A.; Germain, K.; Gore, A.; Breton, B.; Walters-Sen, L. C.; Mullen, T.; Faulkner, N. | 2016 | Fertility and Sterility | 106 | 3(SUPPL.) | e152 |  | Inadequate information |
| High resolution oligonucleotide array-CGH method for preimplantation genetic screening: Preclinical validation and first clinical results | Vallova, V.; Paralova, D.; Smetana, J.; Mikulasova, A.; Hubinka, V.; Slivkova, L.; Zaoralova, R.; Ulicny, B.; Machac, S.; Koudelka, M.; Kuglik, P. | 2013 | Reproductive BioMedicine Online | 26 | SUPPL. 1 | S33-S34 | 10.1016/S1472-6483%2813%2960080-2 | Inadequate information |
| Results from 4 years of preimplantation genetic diagnostics using fluorescent in situ hybridization (fish) method in slovenia | Veble, A.; Volk, M.; Writzl, K.; Dolnicar, B.; Remec, Z.; Kmecl, J.; Valentincic-Gruden, B.; Virant-Klun, I.; Tomazevic, T.; Peterlin, B. | 2009 | Chromosome Research | 17 | SUPPL.1 | S216-S217 | 10.1007/s10577-009-9043-0 | Inadequate information |
| New protocol based on massive parallel sequencing for aneuploidy screening of preimplantation human embryos | Vendrell,; X,; Fernandez, Pedrosa; V,; Trivino,; J.C,; Bautista, Llacer; R,; Collado,; C,; Rodriguez,; O,; Garcia, Mengual; E,; Ferrer,; E,; Calatayud,; C,; Ruiz, Jorro; M, | 2017 | Systems Biology in Reproductive Medicine | 63 | 3 | 162-178 | http://dx.doi.org/10.1080/19396368.2017.1312633 | Wrong study design |
| NGS vs. aCGH for the detection of segmental aneuploidies in human blastocysts | Vera, M.; Michel, C. E.; Mercader, A.; Kokocinski, F.; Rodrigo, L.; Bladon, A. J.; Mateu, E.; Al-Asmar, N.; Blesa, D.; Simon, C.; Rubio, C. | 2014 | Human Reproduction | 29 | SUPPL. 1 | i110 | 10.1093/humrep/29.Supplement_1.1 | Inadequate information |
| Non-Invasive PGS reveals the existence of complementary aneuploidy between DNA obtained from trophectodermbiopsy versus DNA in spent culture medium in the same embryo | Vera-Rodriguez, M.; Diez-Juan, A.; Martinez, S.; Peinado, V.; Mercader, A.; Moreno, I.; Valbuena, D.; Blesa, D.; Simon, C.; Rubio, C. | 2017 | Human Reproduction | 32 | SUPPL. 1 | i14-i15 | 10.1093/humrep/32.Supplement_1.1 | Use of FISH |
| Custom NGS algorithm for consistent and accurate diagnosis of mosaicism in trophectoderm biopsies | Vera-Rodriguez, M.; Navarro, R.; Lopez, P.; Jimenez, J.; Rodrigo, L.; Garcia-Pascual, C. M.; Riboldi, M.; Coprerski, B.; Kayali, R.; Stankewicz, T.; Khajuria, R.; Opeyemi, A.; Yeh, C. S.; Simon, C.; Rubio, C. | 2018 | Reproductive BioMedicine Online | 36 | SUPPL. 1 | e12 |  | Wrong study design |
| Clinical, obstetric and neonatal outcomes of Hungarian preimplantation genetic diagnosis for aneuploidy (PGD-A) cycles of the year 2013 | Vereczkey, A.; Teglas, G. Y.; Margittai, E.; Csenki, M.; Nanassy, L. | 2015 | Human Reproduction | 30 | SUPPL. 1 | i404-i405 | 10.1093/humrep/30.Supplement-1.1 | Outcomes are unclear or not relevant |
| Polar body diagnosis of common aneuploidies by FISH | Verlinsky, Y.; Cieslak, J.; Freidine, M.; Ivakhnenko, V.; Wolf, G.; Kovalinskaya, L.; White, M.; Lifchez, A.; Kaplan, B.; Moise, J.; Valle, J.; Ginsberg, N.; Strom, C.; Kuliev, A. | 1996 | Journal of Assisted Reproduction and Genetics | 13 | 2 | 157-162 |  | Wrong study design |
| Preimplantation diagnosis of common aneuploidies by the first- and second-polar body FISH analysis | Verlinsky, Y.; Cieslak, J.; Ivakhnenko, V.; Evsikov, S.; Wolf, G.; White, M.; Lifchez, A.; Kaplan, B.; Moise, J.; Valle, J.; Ginsberg, N.; Strom, C.; Kuliev, A. | 1998 | Journal of Assisted Reproduction and Genetics | 15 | 5 | 285-289 | 10.1023/A:1022592427128 | Wrong study design |
| Prevention of age-related aneuploidies by polar body testing of oocytes | Verlinsky, Y.; Cieslak, J.; Ivakhnenko, V.; Evsikov, S.; Wolf, G.; White, M.; Lifchez, A.; Kaplan, B.; Moise, J.; Valle, J.; Ginsberg, N.; Strom, C.; Kuliev, A. | 1999 | Journal of Assisted Reproduction and Genetics | 16 | 4 | 165-169 |  | Wrong study design |
| Chromosomal abnormalities in the first and second polar body | Verlinsky, Y.; Cieslak, J.; Ivakhnenko, V.; Evsikov, S.; Wolf, G.; White, M.; Lifchez, A.; Kaplan, B.; Moise, J.; Valle, J.; Ginsberg, N.; Strom, C.; Kuliev, A. | 2001 | Molecular and Cellular Endocrinology | 183 | SUPPL. 1 | S47-S49 | 10.1016/S0303-7207%2801%2900565-2 | Wrong study design |
| Preimplantation testing of polar body aneuploidies in patients of advanced maternal age undergoing in vitro fertilization | Verlinsky, Y.; Kuliev, A. | 1998 | Assisted Reproduction Reviews | 8 | 4 | 223-227 |  | Wrong study design |
| Preimplantation diagnosis for aneuploidies in assisted reproduction | Verlinsky, Y.; Kuliev, A. | 2004 | Minerva Ginecologica | 56 | 3 | 197-203 |  | Wrong study design |
| Chromosomal aneuploidy in embryos conceived with unstimulated cycle IVF | Verpoest, W.; Fauser, B. C.; Papanikolaou, E.; Staessen, C.; Van Landuyt, L.; Donoso, P.; Tournaye, H.; Liebaers, I.; Devroey, P. | 2008 | Human Reproduction | 23 | 10 | 2369-2371 | 10.1093/humrep/den269 | Outcomes are unclear or not relevant |
| Births from embryos with highly elevated levels of mitochondrial DNA | Victor, A.; Griffin, D.; D, K. Gardner; Brake, A.; Zouves, C.; Barnes, F.; Viotti, M. | 2019 | Reproductive BioMedicine Online | 39 | 3 | 403-412 | 10.1016/j.rbmo.2019.03.214 | Comparator is not relevant |
| New insights from one thousand mosaic embryo transfers: features of mosaicism dictating rates of implantation, spontaneous abortion, and neonate health | Viotti, M.; Victor, A.; Barnes, F.; Zouves, C.; Besser, A. G.; Grifo, J. A.; Cheng, E. H.; Lee, M. S.; Lin, P. Y.; Corti, L.; Fiorentino, F.; Spinella, F.; Minasi, M. G.; Greco, E.; Munne, S. | 2020 | Fertility and Sterility | 114 | 3(SUPPL.) | e1-e2 | 10.1016/j.fertnstert.2020.08.029 | Inadequate information |
| Update on the International Society of Mosaic Embryo Transfers | Viotti, M. | 2022 | Fertility and Sterility | 118 | 4(SUPPL.) | e98 | 10.1016/j.fertnstert.2022.08.298 | Inadequate information |
| Validation of copy number variation sequencing for detecting chromosome imbalances in human preimplantation embryos | Wang, L.; Cram, D. S.; Shen, J.; Wang, X.; Zhang, J.; Song, Z.; Xu, G.; Li, N.; Fan, J.; Wang, S.; Luo, Y.; Wang, J.; Yu, L.; Liu, J.; Yao, Y. | 2014 | Biology of Reproduction | 91 | 2 | 37 | http://dx.doi.org/10.1095/biolreprod.114.120576 | Wrong study design |
| IVF embryo choices and pregnancy outcomes | Wang, L.; Wang, X.; Liu, Y.; Ou, X.; Li, M.; Chen, L.; Shao, X.; Quan, S.; Duan, J.; He, W.; Shen, H.; Sun, L.; Yu, Y.; Cram, D. S.; Leigh, D.; Yao, Y. | 2021 | Prenatal Diagnosis | 41 | 13 | 1709-1717 | https://dx.doi.org/10.1002/pd.6042 | Outcomes are unclear or not relevant |
| A strategy using SNP linkage analysis for monogenic diseases PGD combined with HLA typing | Wang, Yuqian; Qin, Meng; Yan, Zhiqiang; Guan, Shuo; Kuo, Ying; Kong, Siming; Nie, Yanli; Zhu, Xiaohui; Zhi, Xu; Qiao, Jie; Yan, Liying | 2020 | Clinical Genetics | 98 | 2 | 138-146 | 10.1111/cge.13770 | Outcomes are unclear or not relevant |
| DOPlify, Target Sequence Enrichment and Allele Drop-Out - is there a benefit? | Warren, K.; Protopsaltis, S.; Jasper, M. | 2019 | Reproductive BioMedicine Online | 38 | SUPPL. 1 | e62 | 10.1016/j.rbmo.2019.03.098 | Inadequate information |
| Validation of a high throughput, low cost NGS PGS assay: Impact of library preparation and read length on resolution | Warren, K.; Wang, P.; Parker, W.; Hodgson, B.; Jasper, M. | 2017 | Human Reproduction | 32 | SUPPL. 1 | i423 | 10.1093/humrep/32.Supplement_1.1 | Wrong study design |
| 4. VALIDATION STUDY OF A NGS-BASED PGT-A WORKFLOW FOR MOSAICISM AND SEGMENTAL COPY NUMBER VARIATION | Wei Wei, J.; Zhang, C.; Qin, X.; Davidson, C. J.; Harris, A.; Rosenwaks, Z.; Xu, K. | 2019 | Reproductive BioMedicine Online | 39 | SUPPL. 1 | e28-e29 | http://dx.doi.org/10.1016/j.rbmo.2019.04.057 | Outcomes are unclear or not relevant |
| Next generation sequencing and PGD | Wells, D. | 2014 | Chromosome Research | 22 | 4 | 594-595 | 10.1007/s10577-014-9437-5 | Inadequate information |
| Next generation sequencing | Wells, D.; Kaur, K.; Fragouli, E.; Munne, S. | 2013 | Reproductive BioMedicine Online | 26 | SUPPL. 1 | S5 | 10.1016/S1472-6483%2813%2960018-8 | Inadequate information |
| Whole-genome sequencing technology for the assessment of embryo genetics and viability | Wells, D.; Kaur, K.; Glassner, M.; Grifo, J.; Fragouli, E.; Munne, S. | 2013 | Fertility and Sterility | 100 | 3(SUPPL.) | S132-S133 | 10.1016/j.fertnstert.2013.07.1596 | Outcomes are unclear or not relevant |
| A novel embryo screening technique provides new insights into embryo biologyandyields the first pregnancies following genomesequencing | Wells, D.; Kaur, K.; Grifo, J.; Anderson, S.; Taylor, J.; Fragouli, E.; Munne, S. | 2013 | Human Reproduction | 28 | SUPPL. 1 | i26 | 10.1093/humrep/det143 | Inadequate information |
| Clinical utilisation of a rapid low-pass whole genome sequencing technique for the diagnosis of aneuploidy in human embryos prior to implantation | Wells, D.; Kaur, K.; Grifo, J.; Glassner, M.; Taylor, J. C.; Fragouli, E.; Munne, S. | 2014 | Journal of Medical Genetics | 51 | 8 | 553-562 | http://dx.doi.org/10.1136/jmedgenet-2014-102497 | Outcomes are unclear or not relevant |
| Validation and clinical application of next generation sequencing for embryo screening: First pregnancy heralds a potential revolution in embryo selection | Wells, D.; Kaur, K.; Rico, A.; Grifo, J.; Taylor, T.; Munne, S. | 2013 | Human Fertility | 16 | 3 | e16-e17 | 10.3109/14647273.2013.810860 | Inadequate information |
| Validation of DNA probes for preimplantation genetic diagnosis (PGD) by fluorescence in situ hybridization (FISH) R1 | Weremowicz, S.; Sandstrom, D. J.; Morton, C. C.; Miron, P. M. | 2006 | Prenatal Diagnosis | 26 | 11 | 1042-1050 |  | Outcomes are unclear or not relevant |
| A prospective, blinded, non-selection study to determine the predictive value of ploidy results using a novel method of targeted amplification based next generation sequencing (NGS) for comprehensive chromosome screening (CCS) | Werner, M. D.; Franasiak, J. M.; Hong, K. H.; Juneau, C. R.; Tao, X.; Landis, J.; Upham, K. M.; Treff, N. R.; Scott, R. T. | 2015 | Fertility and Sterility | 104 | 3(SUPPL.) | e12-e13 |  | Duplicate |
| Targeted NGS provides accurate predictions of segmental (SEG) aneuploidy and prognosticates reduced reproductive potential of the human blastocyst | Werner, M. D.; Goodrich, D.; Tao, X.; Zhan, Y.; Franasiak, J. M.; Juneau, C. R.; Scott, R. T.; Treff, N. R. | 2016 | Fertility and Sterility | 106 | 3(SUPPL.) | e68 |  | Outcomes are unclear or not relevant |
| Induced cellular lysis and increased laser exposure during trophectoderm biopsies can generate low-level mosaic profiles: A prospective study | Whitney, J. B.; Rios, C. M.; Anderson, R. E.; Schiewe, M. C. | 2018 | Human Reproduction | 33 | SUPPL. 1 | i494-i495 | 10.1093/humrep/33.Supplement_1.1 | Inadequate information |
| Combined preimplantation genetic testing for genetic kidney disease: genetic risk identification, assisted reproductive cycle, and pregnancy outcome analysis | Xiao, M.; Shi, H.; Rao, J.; Xi, Y.; Zhang, S.; Wu, J.; Zhu, S.; Zhou, J.; Xu, H.; Lei, C.; Sun, X. | 2022 | Frontiers in Medicine | 9 | 936578 |  | https://dx.doi.org/10.3389/fmed.2022.936578 | Outcomes are unclear or not relevant |
| First birth in Switzerland after array comparative genomic hybridization for chromosomal analysis in polar bodies followed by zygote vitrification and warming | Xie, M.; Oneda, B.; Rauch, A.; Imthurn, B. | 2014 | Chromosome Research | 22 | 4 | 639 | 10.1007/s10577-014-9437-5 | Wrong study design |
| Identification of biparental and diploid blastocysts from monopronuclear zygotes with the use of a single-nucleotide polymorphism array | Xie, P. Y.; Tang, Y.; Hu, L.; Ouyang, Q.; Gu, Y. F.; Gong, F.; Leng, L. Z.; Zhang, S. P.; Xiong, B.; Lu, G. X.; Lin, G. | 2018 | Fertility and Sterility | 110 | 3 | 545 | 10.1016/j.fertnstert.2018.04.034 | Outcomes are unclear or not relevant |
| Reliable high resolution SNP-array analysis of human embryos for genomic's aberration screening and karyomapping | Xiong, R.; Duttagupta, R.; Fung, E.; Levy, B.; Lin, G. | 2015 | Human Reproduction | 30 | SUPPL. 1 | i40 | http://dx.doi.org/10.1093/humrep/30.Supplement-1.1 | Inadequate information |
| Noninvasive chromosome screening of human embryos by genome sequencing of embryo culture medium for in vitro fertilization | Xu, J.; Fang, R.; Chen, L.; Chen, D.; Xiao, J. P.; Yang, W.; Wang, H.; Song, X.; Ma, T.; Bo, S.; Shi, C.; Ren, J.; Huang, L.; Cai, L. Y.; Yao, B.; Xie, X. S.; Lu, S. | 2016 | Proceedings of the National Academy of Sciences of the United States of America | 113 | 42 | 11907-11912 | http://dx.doi.org/10.1073/pnas.1613294113 | Comparator is not relevant |
| Use of next generation sequencing technology is feasible for current PGD with fresh embryo transfer | Xu, K. P.; Victor, A. R.; Zhang, C. H.; Jiang, H.; Li, X. C.; Zhang, X. Q.; Yang, H. M.; Rosenwaks, Z. | 2012 | Reproductive BioMedicine Online | 24 | SUPPL. 2 | S43 | http://dx.doi.org/10.1016/S1472-6483%2812%2960215-6 | Outcomes are unclear or not relevant |
| Whole genome deep sequencing from single cells for preimplantation genetic diagnosis | Xu, K. P.; Victor, A. R.; Zhang, C. H.; Jiang, H.; Zhang, X. Q.; Rosenwaks, Z. | 2011 | Fertility and Sterility | 96 | 3(SUPPL.) | S220 | http://dx.doi.org/10.1016/j.fertnstert.2011.07.847 | Outcomes are unclear or not relevant |
| Detection of chromosome copy number mosaicism in mixed cells by array comparative genome hybridization | Xu, K. P.; Zhang, C. H.; Victor, A. R.; Rosenwaks, Z. | 2012 | Reproductive BioMedicine Online | 24 | SUPPL. 2 | S66-S67 | http://dx.doi.org/10.1016/S1472-6483%2812%2960266-1 | Outcomes are unclear or not relevant |
| Preimplantation genetic testing for carriers of balanced chromosomal rearrangements | Yacuts, O.; Tsukerman, J.; Zhabinskaya, A.; Alekseeva, A.; Volokhanovitch, S.; Tishkevich, O. | 2019 | European Journal of Human Genetics | 27 | SUPPL.1 | 883 | 10.1038/s41431-019-0408-3 | Outcomes are unclear or not relevant |
| Live Bbrth with or without preimplantation genetic testing for aneuploidy | Yan, J.; Qin, Y; Zhao, H.; Sun, Y.; Gong, F.; Li, R.; Sun, X.; Ling, X.; Li, H.; Hao, C.; Tan, J.; Yang, J.; Zhu, Y.; Liu, F.; Chen, D.; Wei, D.; Lu, J.; Ni, T.; Zhou, W.; Wu, K.; Gao, Y.; Shi, Y.; Lu, Y.; Zhang, T.; Wu, W.; Ma, X.; Ma, H.; Fu, J.; Zhang, J.; Meng, Q.; Zhang, H.; Legro, R. S.; Chen, Z.-J. | 2021 | The New England Journal of Medicine | 385 | 22 | 2047-2058 | https://dx.doi.org/10.1056/NEJMoa2103613 | Outcomes are unclear or not relevant |
| Live births after simultaneous avoidance of monogenic diseases and chromosome abnormality by next-generation sequencing with linkage analyses | Yan, L.; Huang, L.; Xu, L.; Huang, J.; Ma, F.; Zhu, X.; Tang, Y.; Liu, M.; Lian, Y.; Liu, P.; Li, R.; Lu, S.; Tang, F.; Qiao, J.; Xie, X. S. | 2015 | Proceedings of the National Academy of Sciences of the United States of America | 112 | 52 | 15964-15969 | https://dx.doi.org/10.1073/pnas.1523297113 | Wrong study design |
| Simultaneous detection of genomic imbalance in patients receiving preimplantation genetic testing for monogenic diseases (PGT-M) | Yang, L.; Xia, J.; Yan, H.; Ding, C.; Shi, Q.; Wu, Y.; Liu, P.; Pan, J.; Zeng, Y.; Zhang, Y.; Chen, F.; Jiang, H.; Xu, Y.; Li, W.; Zhou, C.; Gao, Y. | 2022 | Frontiers in Genetics | 13 |  | 976131 | 10.3389/fgene.2022.976131 | Wrong study design |
| Identification of embryonic chromosomal abnormality using FISH-based preimplantation genetic diagnosis | Ye, Y. H.; Xu, C. M.; Jin, F.; Qian, Y. L. | 2004 | Journal of Zhejiang University - Science | 5 | 10 | 1249-1254 |  | Outcomes are unclear or not relevant |
| Chromosomal complement and clinical relevance of multinucleated embryos in PGD and PGS cycles | Yilmaz, A.; Zhang, L.; Zhang, X. Y.; Son, W. Y.; Holzer, H.; Ao, A. | 2014 | Reproductive BioMedicine Online | 28 | 3 | 380-387 | 10.1016/j.rbmo.2013.11.003 | Outcomes are unclear or not relevant |
| A whole-genome sequencing-based novel preimplantation genetic testing method for de novo mutations combined with chromosomal balanced translocations | Yuan, P.; Xia, J.; Ou, S.; Liu, P.; Du, T.; Zheng, L.; Yin, X.; Xie, L.; Zhang, S.; Yan, H.; Gao, Y.; Zhang, Q.; Jiang, H.; Chen, F.; Wang, W. | 2020 | Journal of Assisted Reproduction and Genetics | 37 | 10 | 2525-2533 | 10.1007/s10815-020-01921-4 | Wrong study design |
| Haploseek: A new 24-hour all-in-one method for combined PGT of monogenic diseases (PGT-M) chromosomal rearrangements (PGT-SR) and aneuploidy (PGT-A) | Zeevi, D. A.; Backenroth, D.; Carmi, S.; Zahdeh, F.; Kling, Y.; Perez, A.; Rosen, T.; Levy-Lahad, E.; Altarescu, G. | 2019 | Reproductive BioMedicine Online | 38 | SUPPL. 1 | e13 | 10.1016/j.rbmo.2019.03.024 | Wrong study design |
| Reliable preimplantation genetic diagnosis in thawed human embryos vitrified at cleavage stages without biopsy | Zhang, L.; Yilmaz, A.; Chian, R. C.; Son, W. Y.; Zhang, X. Y.; Kong, D.; Dahan, M.; Holzer, H.; Tan, S. L.; Ao, A. | 2011 | Journal of Assisted Reproduction and Genetics | 28 | 7 | 597-602 | 10.1007/s10815-011-9556-2 | Outcomes are unclear or not relevant |
| Development and validation of a new next generation sequencing-based protocol to distinguish between balanced translocation and normal chromosomes embryos from robertsonian translocation carriers | Zhao, Q.; Xu, Y.; Yang, K.; Zeng, Y.; Li, R.; Wang, J.; Liu, X.; Xing, L.; Feng, T.; Zhou, C. | 2016 | Human Reproduction | 31 | SUPPL. 1 | i229-i230 | 10.1093/humrep/31.Supplement_1.1 | Inadequate information |
| Application of next-generation sequencing for 24-chromosome aneuploidy screening of human preimplantation embryos | Zheng, H.; Jin, H.; Liu, L.; Liu, J.; Wang, W.-H. | 2015 | Molecular Cytogenetics | 8 | 38 |  | 10.1186/s13039-015-0143-6 | Outcomes are unclear or not relevant |
| 68. PREIMPLANTATION GENETIC TESTING OF MONOGENIC DISEASE: EXPERIENCE IN RUSSIA | Zhikrivetskaya Olegovna, S.; Volkova Leonidovna, Y.; Musatova Valerievna, E.; Sofronova Vladislavovna, Y.; Shirokova Anatolievna, N.; Pomerantseva Alekseevna, E. | 2019 | Reproductive BioMedicine Online | 39 | SUPPL. 1 | e68-e69 | 10.1016/j.rbmo.2019.04.121 | Inadequate information |
| Choice of FISH or array-CGH for PGD of chromosomal rearrangements depending on type, size and position of translocated segments | Zlatopolsky, Z.; Kirillova, I.; Goodman, A.; Ilkevitch, Y.; Rechitsky, S.; Kuliev, A. | 2013 | Reproductive BioMedicine Online | 26 | SUPPL. 1 | S49 | 10.1016/S1472-6483%2813%2960122-4 | Wrong study design |
