# Supplementary material for: A systematic review and meta-analysis of the diagnostic accuracy after preimplantation genetic testing for aneuploidy
Source: PLoS One. 2025 May 14;20(5):e0321859. doi: 10.1371/journal.pone.0321859 (PMC12077728; doi:10.1371/journal.pone.0321859)
Supplement: S3 File — (DOCX) [file pone.0321859.s012.docx]

# S3 File. Cell line supplement results and discussion

## Results of preclinical studies - cell lines

Twenty-two pre-clinical studies met inclusion criteria;[15-37] 15 were full text articles and seven were conference abstracts (S1 Table). Among those included in the meta-analysis, 11 studies used NGS and ten used either CGH, microarray (SNP or aCGH) or quantitative PCR. Thirteen studies obtained their cell lines from Coriell, and 14 studies described the cell line origin, most commonly fibroblasts (n=7). The overall accuracy was 98·8 (95% CI: 96·5-99·6; I^2^=82%), sensitivity 99·1% (95% CI: 96·4-99·8; I^2^=81%), specificity 99·3% (95% CI: 96·6-99·9; I^2^=36%), PPV 99·8% (95% CI: 98·7-100%; I^2^=0%), NPV 97·5 (95% CI: 87·7-99·5%, I^2^=85%) (S1 Fig). The measures of accuracy were nearly identical upon subgroup analyses for NGS or other CCS platforms, and by type of publication (conference abstract vs. full text).

The study quality was low risk of bias across all domains (S2 Table). Fourteen studies reported the cell type (most commonly fibroblasts and lymphocytes), and 18 reported the karyotype of the cell line used (S1 Table). Eight studies created mixtures of cell lines to determine the limit of detection of mosaicism.[18,20,21,24,26,28,31,32] Only seven studies reported the method of genetic confirmation of the cell line, six of which was conventional karyotyping.

## Cell line discussion

Prior to validating embryonic cells, many researchers advocated for the validation of the PGT-A platform to ensure appropriate calibration and accuracy of the test.[128,141] These pre-clinical studies were performed on cell lines, with known karyotype in cell mixtures mimicking a trophectoderm or blastomere biopsy. However, 56% of studies defined the specific cell type and only seven studies investigated the validity of complex chromosome aneuploidy with multiple involved chromosomes. Previous studies have demonstrated that DNA amplification and analysis is impacted by the cell type, where buccal cell DNA degrades rapidly, while fibroblasts more closely resemble blastomeres.[142] Major limitations of this study design are that cell lines are a simplistic model, unlike human embryos, where multiple chromosomes can be involved in non-disjunction and aneuploidy. Validation can be done on blastomeres of donated embryos; however, these studies were excluded, as their actual karyotype cannot be known definitively and should not serve as a gold standard.

**REFERENCES**

141. Biricik A, Cotroneo E, Minasi MG, Greco PF, Bono S, Surdo M, et al. Cross-validation of next-generation sequencing technologies for diagnosis of chromosomal mosaicism and segmental aneuploidies in preimplantation embryos model. Life. 2021;11(4):340. doi: 10.3390/life11040340.

142. Glentis S, Sengupta S, Thornhill A, Wang R, Craft I, Harper JC. Molecular comparison of single cell MDA products derived from different cell types. Reprod Biomed Online. 2009;19(1):89–98.
