# Supplementary material for: A systematic review and meta-analysis of the diagnostic accuracy after preimplantation genetic testing for aneuploidy
Source: PLoS One. 2025 May 14;20(5):e0321859. doi: 10.1371/journal.pone.0321859 (PMC12077728; doi:10.1371/journal.pone.0321859)
Supplement: S4 File — (PDF) [file pone.0321859.s013.pdf]

Systematic review

A list of fields that can be edited in an update can be found here

1. * Review title.

Give the title of the review in English

A systematic review of misclassification after preimplantation genetic testing for aneuploidy

2. Original language title.

For reviews in languages other than English, give the title in the original language. This will be displayed with the English language title.

3. * Anticipated or actual start date.

Give the date the systematic review started or is expected to start.

09/11/2020

4. * Anticipated completion date.

Give the date by which the review is expected to be completed.

30/11/2021

5. * Stage of review at time of this submission.

This field uses answers to initial screening questions. It cannot be edited until after registration.

Tick the boxes to show which review tasks have been started and which have been completed.

Update this field each time any amendments are made to a published record.

The review has not yet started: No

Review stage	Started	Completed
Preliminary searches	Yes	No
Piloting of the study selection process	Yes	No
Formal screening of search results against eligibility criteria	No	No
Data extraction	No	No
Risk of bias (quality) assessment	No	No
Data analysis	No	No

Provide any other relevant information about the stage of the review here.

6. * Named contact.

The named contact is the guarantor for the accuracy of the information in the register record. This may be any member of the review team.

Vanessa Bacal

Email salutation (e.g. "Dr Smith" or "Joanne") for correspondence:

Dr Bacal

7. * Named contact email.

Give the electronic email address of the named contact.

vbaca078@uottawa.ca

8. Named contact address

Give the full institutional/organisational postal address for the named contact.

700-250 Dundas Street West, Toronto, ON, M5T 2Z5

9. Named contact phone number.

Give the telephone number for the named contact, including international dialling code.

416-586-4748

10. * Organisational affiliation of the review.

Full title of the organisational affiliations for this review and website address if available. This field may be

completed as 'None' if the review is not affiliated to any organisation.

University of Toronto

Organisation web address:

https://www.obgyn.utoronto.ca/

11. * Review team members and their organisational affiliations.

Give the personal details and the organisational affiliations of each member of the review team. Affiliation refers to groups or organisations to which review team members belong. **NOTE: email and country now MUST be entered for each person, unless you are amending a published record. PLEASE USE AN INSTITUTIONAL EMAIL ADDRESS IF POSSIBLE.**

Dr Vanessa Bacal. University of Toronto
Dr Urvi Rana. Michigan State University
Dr Crystal Chan. University of Toronto
Dr Rhonda Zwingerman.
Ms Eleni Philippopoulos. Mount Sinai Hospital

12. * Funding sources/sponsors.

Details of the individuals, organizations, groups, companies or other legal entities who have funded or sponsored the review.

2020 Knox Richie Award - Department of Obstetrics and Gynecology, University of Toronto

Grant number(s)

State the funder, grant or award number and the date of award

13. * Conflicts of interest.

List actual or perceived conflicts of interest (financial or academic).

None

14. Collaborators.

Give the name and affiliation of any individuals or organisations who are working on the review but who are not listed as review team members. **NOTE: email and country must be completed for each person, unless you are amending a published record.**

15. * Review question.

State the review question(s) clearly and precisely. It may be appropriate to break very broad questions down into a series of related more specific questions. Questions may be framed or refined using PI(E)COS or similar where relevant.

The objectives of this systematic review are to examine and summarize the evidence around aneuploid

conceptions after PGT-A and euploid ET, and better estimate the false negative
risk after PGT-A and euploid ET.

The second objective of this study is to better understand the risk of a false positive after PGT-A, by evaluating non-selection studies and studies that re-sampled embryos initially

classified as aneuploid or mosaic.

16. * Searches. [1 change]

State the sources that will be searched (e.g. Medline). Give the search dates, and any restrictions (e.g. language or publication date). Do NOT enter the full search strategy (it may be provided as a link or attachment below.)

MEDLINE, PubMed, Embase, CINAHL, Cochrane Central, WHO's International Clinical Trials Registry Platform, ClinicalTrials.gov from inception to December 3, 2020. There were no restrictions on language.

17. URL to search strategy.

Upload a file with your search strategy, or an example of a search strategy for a specific database, (including the keywords) in pdf or word format. In doing so you are consenting to the file being made publicly accessible. Or provide a URL or link to the strategy. Do NOT provide links to your search **results**.

Alternatively, upload your search strategy to CRD in pdf format. Please note that by doing so you are consenting to the file being made publicly accessible.

Do not make this file publicly available until the review is complete

18. * Condition or domain being studied.

Give a short description of the disease, condition or healthcare domain being studied in your systematic review.

Aneuploidy accounts for the majority of miscarriages, as well as congenital anomalies and implantation failure in women. Preimplantation genetic testing for aneuploidy (PGT-A) is a screening test that is applied to embryos created as part of an in vitro fertilization (IVF) cycle. It was developed as a method to optimize implantation and live birth rates, while decreasing miscarriage rates.

19. * Participants/population. [1 change]

Specify the participants or populations being studied in the review. The preferred format includes details of both inclusion and exclusion criteria.

Patients who have undergone PGT-A, or cell lines tested for validation purposes to mimic PGT

20. * Intervention(s), exposure(s). [1 change]

Give full and clear descriptions or definitions of the interventions or the exposures to be reviewed. The preferred format includes details of both inclusion and exclusion criteria.

Preimplantation genetic testing with transfer of euploid, or non-selection embryo transfer (transfer of embryo prior to learning result of genetic testing. We will also include non-transfer of aneuploid embryo. We will also include studies that used cell lines (eg. fibroblasts or lymphocytes) to validate the PGT platform.

21. * Comparator(s)/control. [1 change]

Where relevant, give details of the alternatives against which the intervention/exposure will be compared (e.g. another intervention or a non-exposed control group). The preferred format includes details of both inclusion and exclusion criteria.

Genetic testing of ongoing pregnancies suspected of aneuploidy (amniocentesis, or genetic testing of infant), genetic testing of products of conception after euploid ET with subsequent pregnancy loss, or physical examination of infants.

Rebiopsied (including inner cell mass or whole embryo, with or without trophectoderm rebiopsy) embryo, or aneuploid embryo transferred and genetic testing of products of conception after pregnancy loss or ongoing clinical pregnancy, or physical examination of infants.

22. * Types of study to be included. [2 changes]

Give details of the study designs (e.g. RCT) that are eligible for inclusion in the review. The preferred format includes both inclusion and exclusion criteria. If there are no restrictions on the types of study, this should be stated.

Retrospective or prospective studies including case series, case control studies, cohort studies and randomized controlled trials. We will also include abstracts if full length manuscripts are not available. We will only include studies published in English or French language.

23. Context. [1 change]

Give summary details of the setting or other relevant characteristics, which help define the inclusion or exclusion criteria.

We will exclude case report studies and studies primarily investigating the validity of PGT for monogenic disease or structural arrangements without performing a PGT-A analysis.

We will also exclude studies comparing PGT platforms (eg. array CGH vs NGS) using one of those platforms as the gold standard, where the whole embryo or the inner cell mass isn't resampled.

24. * Main outcome(s).

Give the pre-specified main (most important) outcomes of the review, including details of how the outcome is defined and measured and when these measurement are made, if these are part of the review inclusion

criteria.

True negatives, true positives, positive predictive value, negative predictive value, sensitivity, specificity, positive likelihood ratio, negative likelihood ratio, accuracy or agreement of the two specimen, error rate and discrepancy rate

Measures of effect

Please specify the effect measure(s) for you main outcome(s) e.g. relative risks, odds ratios, risk difference, and/or 'number needed to treat.

Positive predictive value

25. * Additional outcome(s).

List the pre-specified additional outcomes of the review, with a similar level of detail to that required for main outcomes. Where there are no additional outcomes please state ‘None’ or ‘Not applicable’ as appropriate to the review

Sensitivity, specificity, positive predictive value, negative predictive value, positive likelihood ratio, negative likelihood ratio

Measures of effect

Please specify the effect measure(s) for you additional outcome(s) e.g. relative risks, odds ratios, risk difference, and/or 'number needed to treat.

26. * Data extraction (selection and coding). [1 change]

Describe how studies will be selected for inclusion. State what data will be extracted or obtained. State how this will be done and recorded.

We will extract all relevant information from studies that meet final inclusion criteria including: year of publication, country of publication, study design, patient population ((eg. Infertile, RIF, RPL), sample size, primary outcome, method of DNA amplification, Index test: PGT-A platform used, stage of embryo development at biopsy, single vs double embryo transfer, completeness of follow up, Reference standard (eg. ICM biopsy, known karyotype from other cell line, amniocentesis, neonatal exam, type of POC testing (karyotype vs array), type of testing of ongoing pregnancies, key study findings (estimation of error rate). Two independent reviewers will extract and compare the data in duplicate from the selected studies. We will resolve discrepancies by consensus.

27. * Risk of bias (quality) assessment. [1 change]

State which characteristics of the studies will be assessed and/or any formal risk of bias/quality assessment tools that will be used.

Quality of individual studies will be determined using the GRADE approach. For risk of bias, we will use the

QUADAS-2 tool for diagnostic accuracy studies. Both the quality assessment and risk of bias will be implemented by two reviewers independently

28. * Strategy for data synthesis. [2 changes]

Describe the methods you plan to use to synthesise data. This **must not be generic text** but should be **specific to your review** and describe how the proposed approach will be applied to your data. If meta-analysis is planned, describe the models to be used, methods to explore statistical heterogeneity, and software package to be used.

Where data can be synthesized quantitatively, a meta-analysis will be performed using R software, if applicable. Specific outcomes that will be synthesized quantitively include overall accuracy, sensitivity, specificity, negative predictive value and positive predictive values and kappa or phi statistic with 95% confidence intervals. The genetic testing from an ongoing pregnancy or the infant, or the resampled embryo in the event of the initial aneuploidy diagnosis will be considered the reference. Individual embryo data from 2x2 tables from each included study will be used for the meta-analysis. Only studies that perform comprehensive chromosomal screening will be included in the meta-analysis. We will use random effects modeling. I^2 greater than 70% will be considered high statistical heterogeneity.

There will be no minimum number of studies to synthesize. We will assess for studies that contribute high statistical heterogeneity.

Overall accuracy, sensitivity, specificity, negative and positive predictive values, and kappa/phi statistic 80% will be considered excellent agreement.

Validation of euploidy, aneuploidy and mosaic embryos will be analyzed separately. We will conduct a narrative review of the included studies.

29. * Analysis of subgroups or subsets. [2 changes]

State any planned investigation of ‘subgroups’. Be clear and specific about which type of study or participant will be included in each group or covariate investigated. State the planned analytic approach.

We plan on performing a subgroup analysis based on the PGT platform used (microarray, PCR or NGS), reference standard utilized (eg for aneuploidy validation: ICM, whole embryo or known cell lines; for euploidy validation: chorionic villi sampling, amniocentesis, karyotype of infant), stage of embryo at time of biopsy, risk of bias, full text versus abstracts only.

30. * Type and method of review.

Select the type of review, review method and health area from the lists below.

Type of review

Cost effectiveness

No

Diagnostic

Yes

Epidemiologic

No

Individual patient data (IPD) meta-analysis

No

Intervention

No

Living systematic review

No

Meta-analysis

Yes

Methodology

No

Narrative synthesis

Yes

Network meta-analysis

No

Pre-clinical

No

Prevention

No

Prognostic

No

Prospective meta-analysis (PMA)

No

Review of reviews

No

Service delivery

No

Synthesis of qualitative studies

No

Systematic review

Yes

Other

No

Health area of the review

Alcohol/substance misuse/abuse

No

Blood and immune system

No

Cancer

No

Cardiovascular

No

Care of the elderly

No

Child health

No

Complementary therapies

No

COVID-19

No

Crime and justice

No

Dental

No

Digestive system

No

Ear, nose and throat

No

Education

No

Endocrine and metabolic disorders

No

Eye disorders

No

General interest

No

Genetics

Yes

Health inequalities/health equity

No

Infections and infestations

No

International development

No

Mental health and behavioural conditions

No

Musculoskeletal

No

Neurological

No

Nursing

No

Obstetrics and gynaecology

Yes

Oral health

No

Palliative care

No

Perioperative care

No

Physiotherapy

No

Pregnancy and childbirth

No

Public health (including social determinants of health)

No

Rehabilitation

No

Respiratory disorders

No

Service delivery

No

Skin disorders

No

Social care

No

Surgery

No

Tropical Medicine

No

Urological

No

Wounds, injuries and accidents

No

Violence and abuse

No

31. Language.

Select each language individually to add it to the list below, use the bin icon to remove any added in error.
English

There is not an English language summary

32. * Country.

Select the country in which the review is being carried out. For multi-national collaborations select all the countries involved.

Canada

33. Other registration details.

Name any other organisation where the systematic review title or protocol is registered (e.g. Campbell, or The Joanna Briggs Institute) together with any unique identification number assigned by them. If extracted data will be stored and made available through a repository such as the Systematic Review Data Repository (SRDR), details and a link should be included here. If none, leave blank.

34. Reference and/or URL for published protocol.

If the protocol for this review is published provide details (authors, title and journal details, preferably in Vancouver format)

Add web link to the published protocol.

Or, upload your published protocol here in pdf format. Note that the upload will be publicly accessible.

No I do not make this file publicly available until the review is complete

Please note that the information required in the PROSPERO registration form must be completed in full even if access to a protocol is given.

35. Dissemination plans.

Do you intend to publish the review on completion?

Yes

Give brief details of plans for communicating review findings.?

36. Keywords.

Give words or phrases that best describe the review. Separate keywords with a semicolon or new line. Keywords help PROSPERO users find your review (keywords do not appear in the public record but are included in searches). Be as specific and precise as possible. Avoid acronyms and abbreviations unless these are in wide use.

Aneuploidy

Preimplantation genetic testing

PGT

In vitro fertilization

37. Details of any existing review of the same topic by the same authors.

If you are registering an update of an existing review give details of the earlier versions and include a full bibliographic reference, if available.

38. * Current review status.

Update review status when the review is completed and when it is published.New registrations must be ongoing so this field is not editable for initial submission.

Please provide anticipated publication date

Review_Ongoing

39. Any additional information.

Provide any other information relevant to the registration of this review.

40. Details of final report/publication(s) or preprints if available.

Leave empty until publication details are available OR you have a link to a preprint (NOTE: this field is not editable for initial submission). List authors, title and journal details preferably in Vancouver format.

Give the link to the published review or preprint.
